# METTL3/IGF2BP3 axis inhibits tumor immune surveillance by upregulating N^6^-methyladenosine modification of PD-L1 mRNA in breast cancer

**DOI:** 10.1186/s12943-021-01447-y

**Published:** 2022-02-23

**Authors:** Weijun Wan, Xiang Ao, Quan Chen, Yang Yu, Luoquan Ao, Wei Xing, Wei Guo, Xiaofeng Wu, Chengxiu Pu, Xueting Hu, Zhan Li, Mengwei Yao, Donglin Luo, Xiang Xu

**Affiliations:** 1 Department of Stem Cell & Regenerative Medicine, State Key Laboratory of Trauma, Burn and Combined Injury, Daping Hospital, Army Medical University, No. 10, Changjiang Branch Road, Yuzhong District, Chongqing, 400042 China; 2 Department of Breast, Thyroid Surgery, Daping Hospital, Army Medical University, No. 10, Changjiang Branch Road, Yuzhong District, Chongqing, 400042 China

**Keywords:** Breast cancer, PD-L1, m^6^A, METTL3, Immune surveillance

## Abstract

**Background:**

Continual expression of PD-L1 in tumor cells is critical for tumor immune escape and host T cell exhaustion, however, knowledge on its clinical benefits through inhibition is limited in breast cancer. N^6^-methyladenosine (m^6^A) plays a crucial role in multiple biological activities. Our study aimed to investigate the regulatory role of the m^6^A modification in PD-L1 expression and immune surveillance in breast cancer.

**Methods:**

MeRIP-seq and epitranscriptomic microarray identified that PD-L1 is the downstream target of METTL3. MeRIP-qPCR, absolute quantification of m^6^A modification assay, and RIP-qPCR were used to examine the molecular mechanism underlying METTL3/m^6^A/IGF2BP3 signaling axis in PD-L1 expression. B-NDG and BALB/c mice were used to construct xenograft tumor models to verify the phenotypes upon METTL3 and IGF2BP3 silencing. In addition, breast cancer tissue microarray was used to analyze the correlation between PD-L1 and METTL3 or IGF2BP3 expression.

**Results:**

We identified that PD-L1 was a downstream target of METTL3-mediated m^6^A modification in breast cancer cells. METTL3 knockdown significantly abolished m^6^A modification and reduced stabilization of PD-L1 mRNA. Additionally, METTL3-mediated PD-L1 mRNA activation was m^6^A-IGF2BP3-dependent. Moreover, inhibition of METTL3 or IGF2BP3 enhanced anti-tumor immunity through PD-L1-mediated T cell activation, exhaustion, and infiltration both in vitro and in vivo. PD-L1 expression was also positively correlated with METTL3 and IGF2BP3 expression in breast cancer tissues.

**Conclusion:**

Our study suggested that METTL3 could post-transcriptionally upregulate PD-L1 expression in an m^6^A-IGF2BP3-dependent manner to further promote stabilization of PD-L1 mRNA, which may have important implications for new and efficient therapeutic strategies in the tumor immunotherapy.

**Supplementary Information:**

The online version contains supplementary material available at 10.1186/s12943-021-01447-y.

## Background

Currently, breast cancer is the leading commonly diagnosed cancer globally surpassing lung cancer [1]. Although the advances in surgeries, chemotherapy and targeted treatment approaches for breast cancer have improved overall survival rates in recent years, the therapeutic modalities are still limited for aggressive breast cancers such as the triple-negative form [2]. The rapidly growing field of cancer immunotherapy is expanding new horizons for antitumor therapy. Programmed death ligand-1 (also known as B7-H1, CD274 or PD-L1; hereafter referred to as PD-L1) is a 33 kDa type I transmembrane glycoprotein which downregulates T-cell function and cell survival by binding to programmed death-1 (PD-1) receptor; it has gained attraction in recent years [3–5]. PD-L1 inhibits immune-mediated rejection and assists tumor cells to evade the host immune surveillance in the tumor microenvironment [6]. Currently, atezolizumab, a monoclonal antibody that targets PD-L1, was approved in combination with nab-paclitaxel for patients with unresectable locally advanced triple-negative breast cancer (TNBC) or metastatic TNBC expressing PD-L1 [7, 8]. However, in early TNBC, complete pathological response is significantly higher among those who receive immune checkpoint inhibitor plus neoadjuvant chemotherapy, regardless of the PD-L1 levels [9]. Thus, PD-L1 expression can be dynamic [10] during the treatment course and may at least partly explain why some cancer patients with tumors lacking PD-L1 expression can respond favorably to checkpoint inhibitors therapy. Therefore, investigation of upstream regulatory mechanisms of the PD-L1 expression in breast cancer is important for an in-depth understanding of the functions of this immunosuppressive molecule. This could provide us with new strategies to reduce the cellular abundance of PD-L1.

The regulatory mechanisms controlling the PD-L1 expression are complex and multifactorial. These require intensive investigations. STAT, HIF-1, Myc, and other transcriptional factors are known to induce the expression of PD-L1 [11–13]. Besides, recent studies have explored the mechanisms of post-translational modifications on the regulation of PD-L1 expression. For instance, PD-L1 glycosylation triggered by B3GNT3 plays an important role in its expression; deglycosylation increases the anti-PD-L1 antibody binding affinity, leading to more accurate PD-L1 prediction and quantification in clinical outcomes [14]. In addition, poly-ubiquitination of CDK4 and SPOP promotes the degradation of PD-L1 [15], while CSN5 antagonizes its degradation [16]. Acetyltransferase DHHC3-mediated palmitoylation of the cytosolic region promotes PD-L1 expression, and palmitoylation blocks PD-L1 ubiquitination and degradation [17].

Furthermore, epigenetic regulations are also crucial in controlling gene expression. RNA methylation, a type of post-transcriptional modification, has gained widespread attention. As the common principal mRNA methylation type in mammals, N^6^-methyadenosine (m^6^A) modifications are reversible and dynamic; these are regulated by m^6^A “writers”, “erasers”, and “readers” (WERs). They are relevant to RNA fate as modifications manipulate the stability, translation efficiency, regulate alternative polyadenylation, and pre-mRNA splicing [18–21]. Methyltransferase-like 3 (METTL3), a section of the complex m^6^A methyltransferase, is essential for several biological processes, including cell differentiation, proliferation, and survival [22–24]. Recent studies show that m^6^A modification is also important in immunoregulation. METTL3 depletion promotes STAT1 and IRF1 mRNA expression in an m^6^A-YTHDF2-dependent manner, which in turn improves immunotherapeutic response by modulation of tumor-infiltrating cells in the intratumor microenvironment of colorectal cancer [25]. Inhibition of METTL3 weakens PD-1 blockade treatment by altering reprogramming of the bone marrow-derived macrophages [26]. Moreover, FTO-mediated m^6^A modification is implicated in the regulation of melanoma tumorigenesis and resistance to anti-PD-1 therapy [27]. However, the impact of m^6^A regulator(s) on the expression of immune-checkpoint molecules in breast cancer remains unclear and requires further investigation.

In the current study, we identified PD-L1 as a downstream target of METTL3-mediated m^6^A modification in breast cancer cells. Further, insulin-like growth factor 2 mRNA binding protein 3 (IGF2BP3) binding to PD-L1 was verified to recognize the m^6^A modification involving METTL3 stabilized PD-L1 mRNA. Moreover, METTL3 and IGF2BP3 participated in the regulation of tumor immune surveillance. Overall, we found that RNA epigenetic regulation is a novel mechanism of PD-L1 expression regulation in breast cancer. Thus, our study broadened the current molecular understanding of tumor immune surveillance.

## Materials

### Cell culture, generation of the stable cell line, and co-culture experiments

The human breast cancer cell lines (MDA-MB-231, HCC38, SK-BR-3), mouse breast cancer cell line 4T1 and human normal breast epithelial cell lines (MCF10A) were obtained from the American Type Culture Collection (ATCC, Manassas, VA, USA). MDA-MB-231 cells were cultured in L15 (Hyclone, USA) supplemented with 10% fetal bovine serum (FBS, Hyclone, USA). HCC38 and 4T1 were preserved in 1640 medium (Gibco) enhanced with 10% FBS, and SK-BR-3 were grown in McCoys 5A medium (BI, Israel) enhanced with 10% FBS (BI, Israel). 37 °C was used to incubate the cells in a humidified 5% CO_2_ incubator.

For METTL3 and IGF2BP3 knockdown, lentiviral vectors harboring shRNA for knockdown and overexpression of METTL3 and IGF2BP3 and negative control underwent syncretization and the cloned into pLKO.1 vector. The plasmids were transfected using lipofectamine iMAX (Invitrogen, USA) into breast cancer cells according to the manufacturer’s protocol. shRNAs sequences are captured in Additional file 7: Table S1. Briefly, stably transfected cells were selected with 10 μg/ml puromycin (MCE, USA) for 3 weeks.

The peripheral venous blood was collected from healthy volunteers (6 ml) in heparin anticoagulant tube. Ficoll lymphocyte separation solution was added to dilute the blood. The PBMCs isolation from blood was done using gradient density centrifugation and then counted. The MDA-MB-231 cells were supplemented with fresh medium and co-cultured with activated cytokine-induced killer cells for 48 h in a ratio of 1:5 (cancer cell: cytokine-induced killer cells). After incubation, 5 mins was used to centrifuge the plates at 400 g, 5 min. The supernatant was collected and the LDH release assay (Beyotime) was performed based on the instructions from the manufacturer. Absorbance was detected at 490 nm and it was done using Biotek microplate reader. Finally, the supernatant was used for the enzyme-linked immune-sorbent assay (ELISA) for quantifying IFN-γ and IL-2 production (R&D System, Minnesota, USA) as per instructions given by the manufacturer.

### MeRIP-qPCR assay and MeRIP-Seq

The m^6^A immunoprecipitation (MeRIP) procedure was performed according to instructions issued by the manufacturer using a Magna MeRIP™ m^6^A kit (#17–10,499, Merck Millipore, MA). Briefly, purified mRNA was digested by DNase I and then fragmented into ∼100 nt using RNA fragmentation reagent and incubated at 94 °C. After fragmenting, the stop buffer was added, following which standard ethanol precipitation was performed and collected. The anti-m^6^A antibody for 12 μg was pre-incubated with 50 μl beads in IP buffer (150 mM NaCl, 0.1% NP-40, 10 mM Tris–HCl, pH 7.4) at room temperature for 1 h. Next, 6 μg of fragment mRNAs were added to the antibody-beads mixture and incubated at 4 °C for 4 h on a rotator. After adequate washing, immunoprecipitated mixture was digested using high concentration of proteinase K, and the bound RNAs were extracted using phenol-chloroform method and ethanol precipitation and were used for qPCR analysis or library construction. qPCR analysis determined the modification of m^6^A in PD-L1 analysis based on precise primers (primers for MeRIP-qPCR were listed in Additional file 7: Table S1). All m^6^A sites of PD-L1 were predicted using SRAMP (http://www.cuilab.cn/sramp) [51]. We created primers to make sure that the target sequence included all these sites within 100 nt length. SMARTer smRNA-Seq Kit was used to perform the library constructions of IP-RNA samples with a small fraction of fragmented mRNAs used as input, and sequenced on the Illumina HiSeq X Ten platform.

### Epitranscriptomic microarray analysis

Briefly, the total RNAs were immunoprecipitated using anti-N6-methyadenosine (m^6^A) antibody. The elute of immunoprecipitation magnetic beads was called “IP” which is m^6^A modified RNAs. “Sup” was the recovered supernatant which is unmodified RNAs. We labeled the “Sup” and “IP” RNA used for Cy5 and Cy3, respectively, as cRNAs. Hybridization of cRNA was done after merging to Arraystar Human m^6^A Epitranscriptomic Microarray (8 × 60 K, Arraystar, Rockville, MD, USA). Finally, the array was scanned using an Agilent scanner G2505C.

### RNA immunoprecipitation (RIP)

RIP assay was performed using Magna RIP Kit (17–700, Millipore, MA) according to the instructions by the manufacturer. Briefly, 5 μg anti-METTL3 (Abcam, USA), anti-IGF2BP3 (Millipore, Germany) or anti-N^6^-methyladenosine (m^6^A) (Millipore, Germany) and anti-rabbit IgG (Millipore, Germany) were incubated with 50 μL magnetic beads before cell lysates were added (approximately 2 × 10^7^ cells per sample). Then, the RNA-protein IP complexes were washed 6 times and proteinase K digestion buffer was used for incubation to remove the proteins. Finally, RNAs were extracted by phenol-chloroform RNA extraction and purified for qPCR analysis. Normalization of the relative enrichment was done to the input as: %Input =1/10 × 2^Ct [IP] – Ct [input]^.

### RNA total m^6^A quantification

EpiQuik™ m^6^A RNA Methylation Quantification Kit (Colorimetric) (Epigentek, USA) was used to colorimetrically measure the total level of m^6^A in breast cancer cells. Two hundred nanogram RNA was briefly combined with the capture antibody in each well which was used for subsequent detection. During multiple incubations, colorimetric measurement of m^6^A content was done at 450 nm wavelength and calculated based on the standard curve.

### RNA stability assay

Breast cancer cells were treated as follows: 6-well plates were used to seed the cells overnight, and actinomycin D (5 μg/mL, HY-17559, MedChemExpress) was used to treat them for 0, 2, 4, 6 h. Total RNA was isolated using TRIzol and quantified by qRT-PCR. Group expression of the mRNA at the indicated times was calculated and normalized by GAPDH.

### Luciferase reporter assay

cDNAs with firefly luciferase which contained full-length sequence of PD-L1 were cloned into pGL3-control vectors (Promega). For mutant 1, 2, 3, and 1–3 reporter plasmids, cytosine (C) replaced marked adenosine (A) in m^6^A motif. Pre-treated breast cancer cells were seeded into 6-well plates followed by co-transfection with 0.5 μg of wild-type or mutated PD-L1 reporter plasmids with 25 ng pRL-TK plasmids (renilla luciferase reporter vector) using jetPRIME Polyplus kit. After 24–36 h, cells were harvested and the luciferase activity was assessed using Dual-Glo Luciferase system (Promega). This was normalized to pRL-TK activity. Each experiment was conducted in triplicates.

### Quantitative real-time PCR

TRIzol Reagent (Invitrogen, USA) was used to extract total RNA from cells as per instructions of the manufacture. The mRNA levels were examined using SYBR Premix Ex Taq (Takara, Dalian, China). GAPDH normalized the results and quantification of the mRNAs relative expression was done using the 2^–∆∆Ct^ method. Used primers are presented in Additional file 7: Table S1.

### Western blot

Extraction of total protein from breast cancer cells using pre-chilled RIPA buffer (Beyotime, Shanghai, China). After protein quantification, sample of protein of equal amounts were loaded and were 10% SDS-PAGE was applied to separate them and then moved onto 0.45 μm PVDF membranes (Millipore, USA). After blocking by 5% non-fat milk in TBST for 1.5 h, incubation of the membranes was done at 4 °C overnight using the corresponding primary antibodies. Then, secondary antibodies were applied in the incubation under room temperature for 1 h after being washed thrice with TBST. Detection of immunoblots was done using an imaging system (Bio-Rad, USA). Each antibody used in this study is captured in the Additional file 7: Table S1.

### Flow Cytometry

PBS was used to wash harvested cells and they were incubated for 30 min on ice using 2% FBS and appropriate antibodies in PBS. After washing with PBS, the samples were analyzed on LSRFortessa SORP (BD Biosciences, Franklin Lakes, NJ), and data was done in FlowJo (Ashland, OR).

### Immunohistochemistry

Paraffin was used to embed Xenograft tumors and then they were cut into sections of 4 μm. Section. Staining of TMA was done using eosin, hematoxylin, or incubated with primary antibodies, with the aid of ElivisionTM plus Polymer HRP immunohistochemistry kit (Maxim, Fujian, China). Capturing of images of representative fields was done on Aperio ImageScope (Leica Biosystems, Wetzlar, Germany).

### Absolute quantification of m^6^A modification

For detection of absolute m^6^A levels, the probes L1 (left probe) and R1 (right probe) were designed. Each probe contained a universal primer-specific sequence used for PCR amplification (red and blue) and a target-specific sequence (orange and green), which were complementary to the RNA target immediately upstream and downstream of the m^6^A site, respectively. Although probes L1 and R1 would hybridize adjacent to each other with the RNA around the m^6^A site, they could not be ligated with T3 DNA ligase which has lesser selectivity with m^6^A modification, reflecting the non-methylated RNA levels at this site by this way. To quantitatively determine the m^6^A modification fraction in RNA transcript, the non-m^6^A site of the same RNA transcript was selected as the reference site, as it only contains “A”. The probes L2 and R2, which were RNA complementary target immediately upstream and downstream of the non-m^6^A site, were respectively designed. Where non-m^6^A, the probes L2 and R2 would be lowered RNA transcript expression levels could be detected by PCR. Finally, the m^6^A modification fraction could be precisely determined by real-time fluorescence PCR signals at the m^6^A site and non-m^6^A site. The schematic diagram is captured in Additional file 5: Fig. S2 g. Primers used are listed in Additional file 7: Table S1.

### Animals

B-NDG mice (4–6 weeks old, female) were supplied by Jiangsu Biocytogen Laboratory Animal Co. (BCM002F). BALB/c mice (4 weeks old, female) were supplied by the Army Medical University, Chongqing and Precision Biotech. Army Medical University and institutional review board of Daping Hospital approved all the procedures. For subcutaneous xenograft experiments in B-NDG mice, approximately 1 × 10^6^ MDA-MB-231 and there was subcutaneous injection of the cells that resuspended in 100 μl PBS into the left flank of the mice and were divided into 11 groups randomly (each containing 5 mice). After the treatment as shown in Additional file 5: Fig. S4a, Atezolizumab (Selleck, Shanghai, China) or corresponding iso control antibody (Selleck, Shanghai, China) was injected intratumorally on day 3, 6, 9, 12, 15 post-MDA-MB-231 inoculations, and 5 × 10^6^ cytokine-induced killer (CIK) cells were injected in the tail vein on day 7, 14, 21. Tumor sizes were measured every 2–3 days. After the feeding, tumors of the mice were removed through sacrifice. Recording of tumor weight was done as well as volume estimation according to the formula: 1/2 × (length×width^2^).

For peritoneal- and subcutaneous-tumor xenograft models in BALB/c mice, approximately 1 × 10^6^ 4T1 cells ([4T1, sh-control, sh-control+ISO mAb, sh-METTL3–1#, sh-METTL3–2#, sh-control+PD-L1 mAb] and 4T1 cells expressing the corresponding luciferase), per mouse suspended in 100 μl PBS were intraperitoneal injected or subcutaneously injected in the flank, respectively. The iso control and PD-L1 mAb (Bio X Cell, Beijing, China) were conducted by intraperitoneal injection (150 μg/mouse) on day 3-, 6-, 9-, 12-, 15-, 18-post-4T1 cells inoculation. FUSION FX imaging system (Vilber Lourmat, Paris, France) was used to perform bioluminescence imaging of tumor-bearing mice to evaluate the tumor growth. All the mice were monitored for survival and tumor volume. Survival was based on the number of days from the inoculation tumor cell to the day of animals were to be euthanized based on the following symptoms: hemiparesis, seizures, loss of weight (more than 20%), inability to move, and other serious neurological deficits symptoms. The results were analyzed using GraphPad Prism 7.0 software. Mice were anesthetized and sacrificed; tumors obtained were processed for IHC staining.

### Statistical analysis

GraphPad Prism 7.0 (GraphPad Software, La Jolla, CA, USA) was applied to analyze the data. Results are presented in the form of means ± SD not less than three biological replicates. Student’s t-tests were used to compare between two groups and one-way analysis of variance (ANOVA) and for comparison of multiple groups was done using Dunnett’s test. Pearson correlation analysis was done to find the correlation between PD-L1 and METTL3 or IGF2BP3 expression levels. *P*-value less than 0.05 in all the tests was taken to be statistically significant.

## Results

### PD-L1 expression is regulated by METTL3-mediated m^6^A modification in breast cancer

**Fig. 1:**
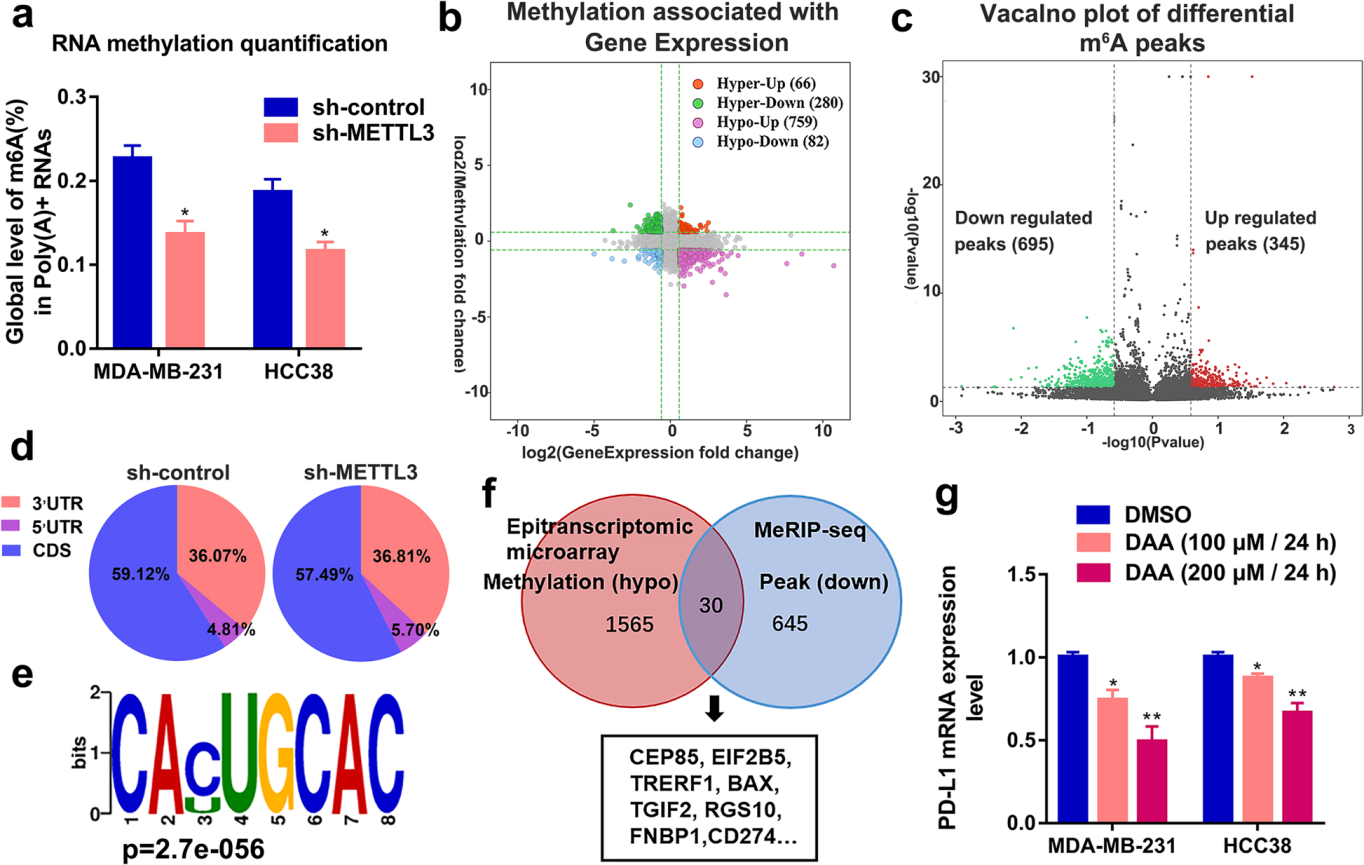
PD-L1 is a downstream target of METTL3. **a** The global content of m^6^A was examined by RNA methylation quantification assay. **b** The starplot presented the distribution of genes with both differential (hyper or hypo) methylation level (Y axis; |fold change| ≥ 1.5) and differential (up or down) gene expression level (X axis; |fold change| ≥ 1.5) in sh-METTL3 with control groups. **c** Volcano plot of changed m^6^A peaks was identified by MeRIP-seq in control and METTL3-knockdown MDA-MB-231cells. **d** Distribution of total m^6^A peaks in sh-control and sh-METTL3 groups were shown. **e** Top sequence motif was identified from MeRIP-seq. **f** Venn diagram showed the down-modified genes following METTL3 knockdown. **g** The mRNA expression levels of MDA-MB-231 and HCC38 cells were tested by qRT-PCR after 3-deazaadenosine (DAA) treatment in the indicated concentration. *p < 0.05; **p < 0.01

To investigate the regulatory role of m^6^A modification in the post-transcriptional expression of PD-L1, we first used the m^6^A target database (http://m6a2target.canceromics.org/#/) [52], based on the results of diverse high-throughput sequencing and mass spectrometry, to obtain information on m^6^A modification in PD-L1. Predicted results showed that PD-L1 could be modified by METTL3-mediated m^6^A modification. Since METTL3 is an important m^6^A writer, we further examined whether METTL3 exerted regulatory effects on the overall m^6^A modification in the breast cancer cells. Indeed, the knockdown of METTL3 reduced the global m^6^A modification in MDA-MB-231 and HCC38 cells in the RNA methylation quantification assay (Fig. 1a). Additionally, the results from m^6^A epitranscriptomic microarray showed that 346 genes were hyper-methylated while 841 genes were hypo-methylated, following METTL3 inhibition as compared to the control group (Fig. 1b). The 695- and 345-m^6^A peaks represented the total statistical decrease and increase in the MeRIP-seq results, respectively, in METTL3 knockdown cells relative to the control (Fig. 1c). Subsequent investigation on the m^6^A peak distributions revealed that total m^6^A distribution patterns were the same in the METTL3 knockdown and control groups (Fig. 1d). The consensus motif which was highly concentrated in m^6^A sites was present in both the control and METTL3 knocked-down cells (Fig. 1e). At the intersection of findings from the epitranscriptomic microarray and MeRIP-seq, 30 sequences from 24 genes that harbored both hypo-methylation and down-regulated peaks were identified, including PD-L1 (CD274) (Fig. 1f). In addition, the methylation inhibitor, 3-deazaadenosine (DAA), also significantly decreased PD-L1 expression in a dose-dependent manner (Fig. 1g). Furthermore, the interaction differences between METTL3 and PD-L1 were also observed in RIP-seq data from sh-METTL3-MDA-MB-231 cells (Additional file 1: Fig. S1a). These results indicated that METTL3 is involved in the regulation of m^6^A modification of PD-L1 mRNA in breast cancer cells. Due to the higher expression of PD-L1 mRNA, MDA-MB-231, HCC38, SK-BR-3, and 4T1 cell lines were chosen for subsequent experiments (Additional file 1: Fig. S1b).

### METTL3 increases m^6^A modification and expression of PD-L1 mRNA

**Fig. 2:**
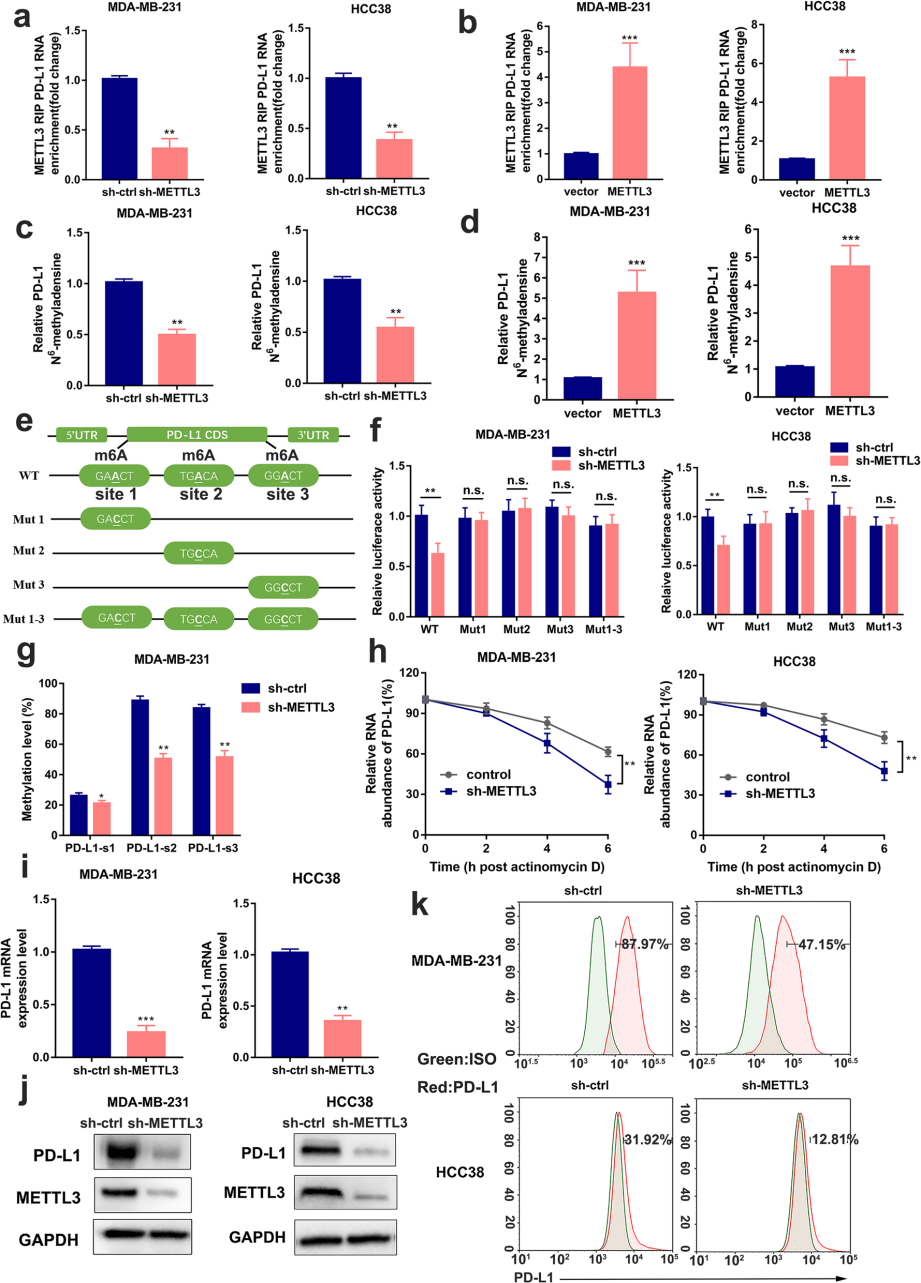
METTL3 increases m^6^A modification and expression of PD-L1 mRNA. **a-b** The interaction between METTL3 and PD-L1 mRNA was analyzed by RIP-qPCR assay in MDA-MB-231 and HCC38 cells with METTL3 knockdown or overexpression. **c-d** The relative levels of m^6^A in PD-L1 were tested by MeRIP-qPCR from MDA-MB-231 and HCC38 cells with overexpression or knockdown of METTL3. **e** Putative m^6^A modification sites in the CDS sequence of PD-L1 and synonymous mutations in the PD-L1 CDS. **f** Relative activity of the WT or Mut luciferase reporters in METTL3-silenced MDA-MB-231 and HCC38 cells was determined (normalized to negative control groups). **g** The m^6^A levels of three specific sites of PD-L1 (correspond to the figure e) were determined by absolute quantification of m^6^A modification. **h** PD-L1 mRNA levels were determined by qRT-PCR in MDA-MB-231 and HCC38 cells (control and METTL3 disruption) after actinomycin D treatment (normalized to 0 h). **i-k** PD-L1 mRNA, protein and cell surface expression levels were detected by qRT-PCR, western blot and flow cytometry in sh-Ctrl or sh-METTL3 MDA-MB-231 and HCC38 cells. Values are the mean ± SD of three independent experiments. *p < 0.05; **p < 0.01; ***p < 0.001; n.s., no significance

To examine the role of METTL3 in post-transcriptional modification of PD-L1, using the RIP-qPCR assay, we observed a significantly higher METTL3 enrichment with PD-L1 mRNA relative to IgG control (Additional file 2: Fig. S2a). Silencing METTL3 suppressed this enrichment in MDA-MB-231, HCC38, SK-BR-3, and 4T1 cells, while overexpression of METTL3 resulted in higher enrichment with PD-L1 mRNA (Fig. 2a, b, Additional file 2: Fig. S2b, c). Furthermore, consistent with the results of the MeRIP-seq, a significant decrease in m^6^A modified PD-L1 was observed by MeRIP-qPCR assay upon METTL3 disruption and METTL3 overexpression increased the levels of m^6^A modified PD-L1 mRNA in MDA-MB-231, HCC38 (Fig. 2c, d), SK-BR-3, and 4T1 cells (Additional file 2: Fig. S2d, e).

To enhance the understanding of the role of m^6^A modification in the regulation of PD-L1 expression, the online tool SRAMP (http://www.cuilab.cn/sramp) [51] was used to predict m^6^A sites, and we constructed a wild-type (WT) and four mutant- (Mut1, 2, 3, 1–3) plasmids to examine the specific modifications of PD-L1. The wild-type plasmid consisted of the full-length CDS sequence with intact m^6^A sites, while each of the mutants had A-C mutations which eliminated the impact of m^6^A methylation (Fig. 2e). As shown in Fig. 2f, the relative luciferase activity of WT remarkably reduced upon METTL3 knockdown, but those for Mut groups were resistant to the effect of METTL3 silencing. To determine the m^6^A methylation levels at each site, we used absolute quantification in the m^6^A modification assay. This is a new and ultrasensitive quantitation assay for the accurate determination of m^6^A at single-nucleotide resolution. Its schematic diagram is shown in Additional file 2: Fig. S2 g. We found that METTL3 could significantly methylate PD-L1 mRNA especially on sites 2 and 3 (Fig. 2g). Furthermore, we found the knockdown of METTL3 led to lower mRNA stability owing to the reduced half-life of PD-L1 transcript after treatment with actinomycin D (Fig. 2h, Additional file 2: Fig. S2f). Collectively, these results suggested that METTL3 directly interacted with PD-L1 and could regulate m^6^A modification of PD-L1 mRNA.

Additionally, PD-L1 mRNA and protein expression levels decreased upon METTL3 knockdown in MDA-MB-231, HCC38 (Fig. 2i, j), SK-BR-3, and 4T1 cells (Additional file 2: Fig. S2h, i). We also determined the cell-surface PD-L1 expression by flow cytometry as shown in Fig. 2k and Additional file 2: Fig. S2j. Taken together, our results suggested that PD-L1 expression was regulated by METTL3-mediated m^6^A modification.

### IGF2BP3 mediates PD-L1 mRNA expression in an m^6^A-dependent manner

**Fig. 3:**
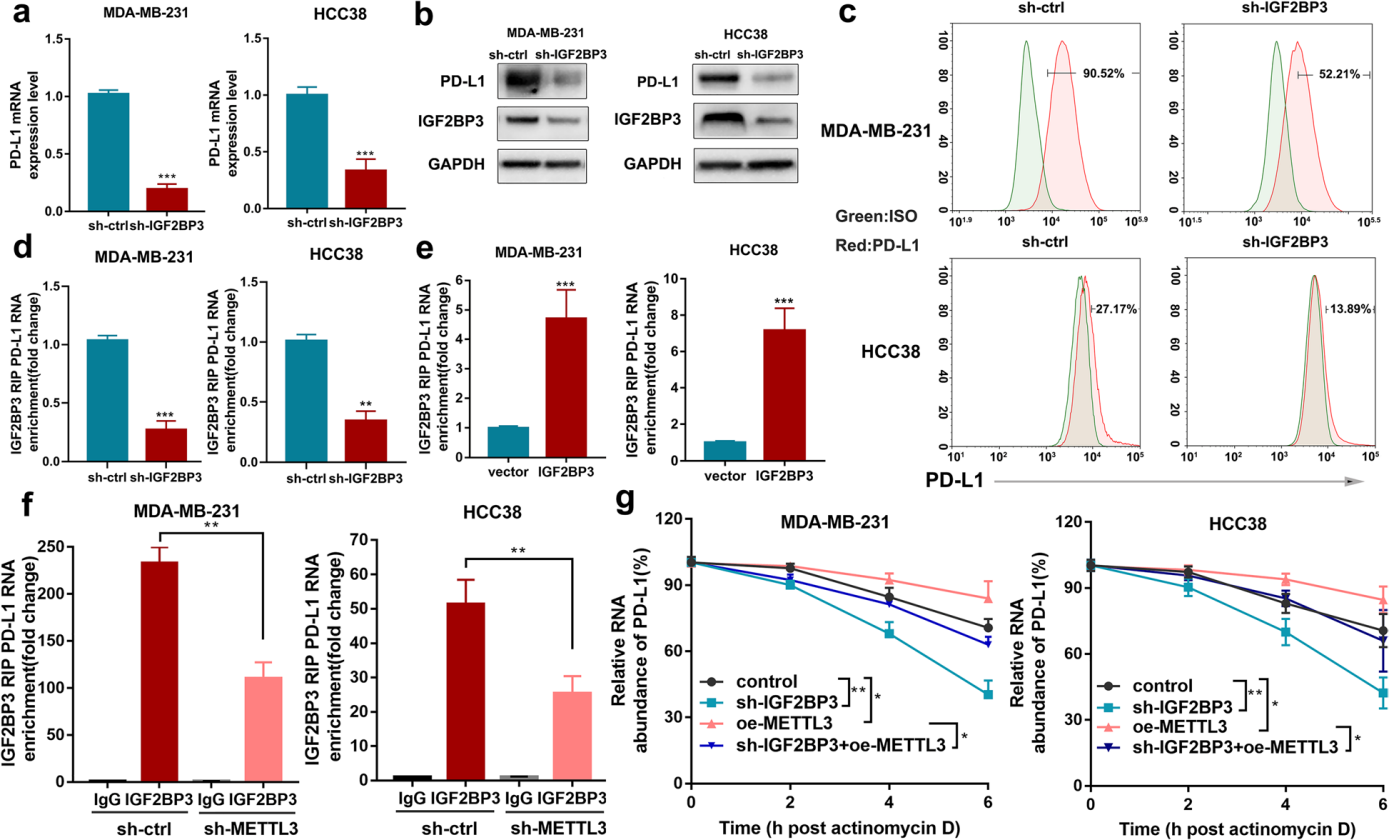
IGF2BP3 mediates PD-L1 mRNA expression in an m^6^A-dependent manner. **a-c** PD-L1 mRNA, protein and cell surface expression levels were tested by qRT-PCR, western blot and flow cytometry in sh-ctrl or sh-IGF2BP3 MDA-MB-231 and HCC38 cells. **d-e** The interaction between IGF2BP3 and PD-L1 mRNA was analyzed by RIP-qPCR assay with overexpression or knockdown of IGF2BP3. **f** The binding of IGF2BP3 was tested by RIP-qPCR in sh-METTL3 and control cells. **g** PD-L1 mRNA levels were analyzed by qRT-PCR assay in MDA-MB-231 and HCC38 cells after actinomycin D treatment. Results were presented as mean ± SD of three independent experiments. *p < 0.05; **p < 0.01; ***p < 0.001

Recently, the regulatory effects of “m^6^A readers” on m^6^A-modified transcripts were confirmed. IGF2BPs, including IGF2BP1/2/3, are a distinct family of m^6^A readers that can recognize and bind to thousands of mRNA transcripts targets through the m^6^A motif. These play a crucial role in mRNA stabilization [44]. To investigate the role of IGF2BPs in the modulation of PD-L1 mRNA, IGF2BP1–3 was knocked down in breast cancer cells, and our results showed that PD-L1 expression was significantly inhibited by IGF2BP3 knockdown (Fig. 3a, Additional file 3: Fig. S3b) instead of IGF2BP1/2 disruption (Additional file 3: Fig. S3a). Due to the lower expression of IGF2BP3 in 4T1 cells, we used MDA-MB-231, HCC38, and SK-BR-3 cells to investigate the regulatory mechanism of IGF2BP3. The total protein and membrane expressions of PD-L1 were remarkably reduced upon IGF2BP3 disruption in breast cancer cells (Fig. 3b, c, Additional file 3: Fig. S3c, d). To confirm whether IGF2BP3 was a potential reader of PD-L1 m^6^A methylation, direct binding interaction between IGF2BP3 and PD-L1 mRNA was evaluated by RIP-qPCR assay compared with IgG (Additional file 3: Fig. S3e). The results also showed that PD-L1 mRNA levels increased upon IGF2BP3 overexpression and decreased when IGF2BP3 was knocked down in breast cancer cell lines (Fig. 3d, e, Additional file 3: Fig. S3f, g). As shown in Additional file 3: Fig. S3h, m^6^A mutant sites in the PD-L1 transcript limited the binding of IGF2BP3. Moreover, METTL3 knockdown inhibited the binding interaction between IGF2BP3 and PD-L1 mRNA (Fig. 3f, Additional file 3: Fig. S3i), which suggested that IGF2BP3 could bind PD-L1 mRNA in a METTL3/m^6^A-dependent manner.

In addition, IGF2BP3 disruption reduced the stability of PD-L1 mRNA, and the knockdown of IGF2BP3 could reverse the increased PD-L1 stability mediated by METTL3 overexpression (Fig. 3g, Additional file 3: Fig. S3j). Moreover, western blotting results showed that overexpression of METTL3 and IGF2BP3 led to a substantial increase in PD-L1 expression, while IGF2BP3 disruption could reverse this effect. The PD-L1 expression significantly decreased due to METTL3 and IGF2BP3 deficiency; however, IGF2BP3 overexpression could not rescue the PD-L1 expression levels upon METTL3 knockdown (Additional file 3: Fig. S3k). Taken together, these results demonstrated that PD-L1 mRNA stability and expression were upregulated through the METTL3-IGF2BP3 axis in an m^6^A-dependent manner.

### METTL3/IGF2BP3-downregulated antitumor immunity in breast cancer cells

**Fig. 4:**
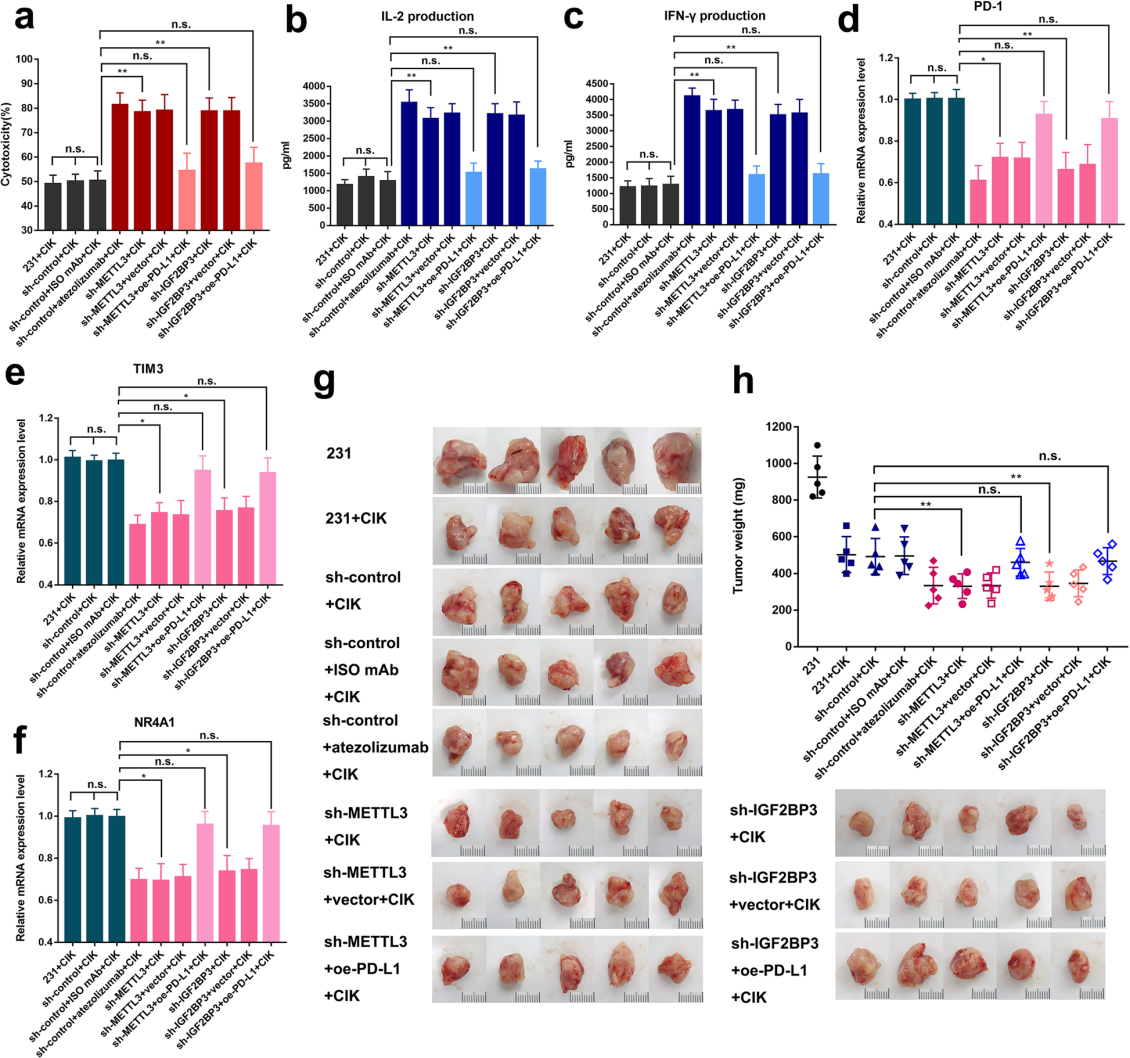
METTL3/IGF2BP3-downregulated antitumor immunity in breast cancer cells. **a** The cytotoxicity was measured by lactate dehydrogenase (LDH) release assay after incubation for 48 h. **b-c** The IFN-γ and IL-2 protein levels in co-culture medium were measured by ELISA after 48 h co-incubation. **d-f** The PD-1, TIM3 and NR4A1 mRNA expression levels were tested by qRT-PCR. **g** Images at the end points of subcutaneous xenograft tumors formed by MDA-MB-231 cells in B-NDG mice (*n* = 5 for each group; scale bar, 1 cm). **h** Tumors weight were measured in the xenograft mice. Results were presented as mean ± SD of three independent experiments. *p < 0.05; **p < 0.01; n.s., no significance

Tumor-derived PD-L1 exerts significant inhibition on antitumor T-cell activation. To investigate the role of METTL3 or IGF2BP3 in PD-L1-mediated tumor immune surveillance, the cytokine-induced killer (CIK) cells were co-cultured with MDA-MB-231 cells. LDH release assay showed that METTL3-, IGF2BP3-knockdown and atezolizumab (anti-PD-L1) treatment increased the breast cancer cell sensitivity towards T-cell killing as compared to control groups, and these effects could be reversed by the overexpression of PD-L1 (Fig. 4a). In addition, T cells were activated with the increase in IL-2 and IFN-γ secretion due to METTL3 or IGF2BP3 knockdown, and by atezolizumab treatment; overexpression of PD-L1 could reverse these effects (Fig. 4b, c). Consistent with PD-L1 binding to its receptor on activated T lymphocytes resulting in T cell exhaustion, we also detected the expression of exhaustion markers, including PD-1, TIM3, and NR4A1, in the T-cells isolated from co-cultured medium. As shown in Fig. 4d-f, we found that PD-1, TIM3, and NR4A1 mRNA levels reduced significantly upon METTL3 or IGF2BP3 deficiency in MDA-MB-231 cells; overexpression of PD-L1 could reverse these effects. Our results suggested that PD-L1 could be a key mediator of METTL3/IGF2BP3-induced tumor immune surveillance in breast cancer cells.

To examine whether METTL3 or IGF2BP3 inhibition–mediated suppression of tumor progression was dependent on PD-L1 in vivo, we used MDA-MB-231 cells (stable METTL3-knockdown, IGF2BP3-knockdown, and simultaneous PD-L1 overexpression [rescue condition]) and subcutaneously injected them into female B-NDG mice for tumor xenograft. Following the treatment as described above (Additional file 4: Fig. S4a), indeed the silencing of METTL3 or IGF2BP3 could suppress tumor growth (Fig. 4g), tumor weight, and volume, similar to effects observed upon PD-L1 blockade treatment; PD-L1 overexpression was able to partially rescue tumor growth (Fig. 4g, h, Additional file 4: Fig. S4b, c). These results indicated that the knockdown of METTL3/IGF2BP3 could enhance T cell-mediated antitumor immunity to alleviate breast cancer progression by downregulating PD-L1 expression.

### METTL3 knockdown enhances antitumor immunity and immune infiltration

**Fig. 5:**
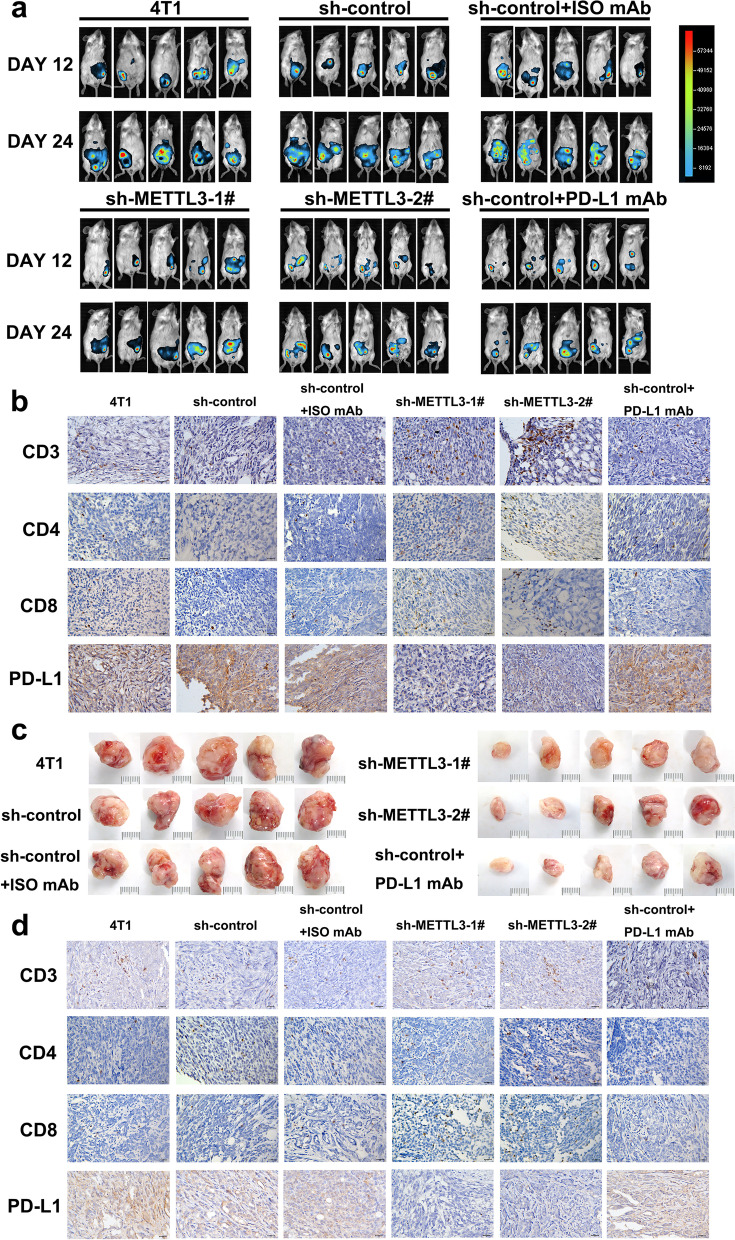
METTL3 knockdown enhances antitumor immunity and immune infiltration. **a** Bioluminescent images of intraperitoneal xenografted tumors from the indicated groups for Day 12 and Day 24. **b** The CD3^+^, CD4^+^ and CD8^+^ densities and PD-L1 expression were determined by Immunohistochemical analysis in the intraperitoneal xenografted tumors. Scale bar, 20 μm. **c** Images of subcutaneous tumors from the indicated groups (scale bar: 1 cm). **d** The CD3+, CD4+ and CD8+ densities and PD-L1 expression were determined by Immunohistochemical assay in the subcutaneous xenograft models. Scale bar, 20 μm

Due to the low expression of IGF2BP3 in 4T1 cells, we constructed stable METTL3-knockdown 4T1 cell lines. To evaluate the immune regulatory effect of METTL3, a syngeneic 4T1 murine tumor model was constructed with immune-competent female BALB/c mice through peritoneal and subcutaneous tumors xenografts. First, the two METTL3-knockdown 4T1 cells were stably engineered to express luciferase for bioluminescence imaging in vivo. The bioluminescence imaging was performed at 12 and 24 days after transplantation. We found that intraperitoneally xenografted tumors in METTL3-silenced groups grew more slowly and prolonged the overall survival as compared to those in the control group, consistent with PD-L1 mAb treatment (Fig. 5a, Additional file 4: Fig. S4d, e). A concomitant decrease in PD-L1 expression was observed due to METTL3 deficiency; higher densities of CD3^+^, CD8^+^, and CD4^+^ T-cell infiltrations were also found in sh-METTL3–1/2# tumors as compared to control tumors which were validated by IHC staining (Fig. 5b, Additional file 4: Fig. S4f). As the therapeutic efficacy of PD-L1 mAb is dependent on the blockade of PD-1/PD-L1 interaction, we speculated that PD-L1 expression in each group was not significantly different. Additionally, in the subcutaneous xenograft models, tumoral METTL3 inhibition and anti-PD-L1 therapy could both markedly limit tumor growth of the mice as compared to the control groups (Fig. 5c, Additional file 4: Fig. S4g, h). The tumors progression were inhibited due to transplanted METTL3-knockdown 4T1 cells showed low PD-L1 levels. Consistent with the results of the intraperitoneal xenografted tumors, METTL3 disruption in transplanted tumors increased CD3^+^, CD4^+^, and CD8^+^ T-cell infiltrations (Fig. 5d, Additional file 4: Fig. S4i). These results suggested the METTL3 was involved in the tumor immune escape by upregulation of PD-L1 expression which inhibited intertumoral T-cell infiltration; tumoral METTL3 deficiency showed similar responses to those upon PD-L1 blockade treatment.

### PD-L1 expression positively correlates with METTL3 and IGF2BP3 expression in breast cancer

**Fig. 6:**
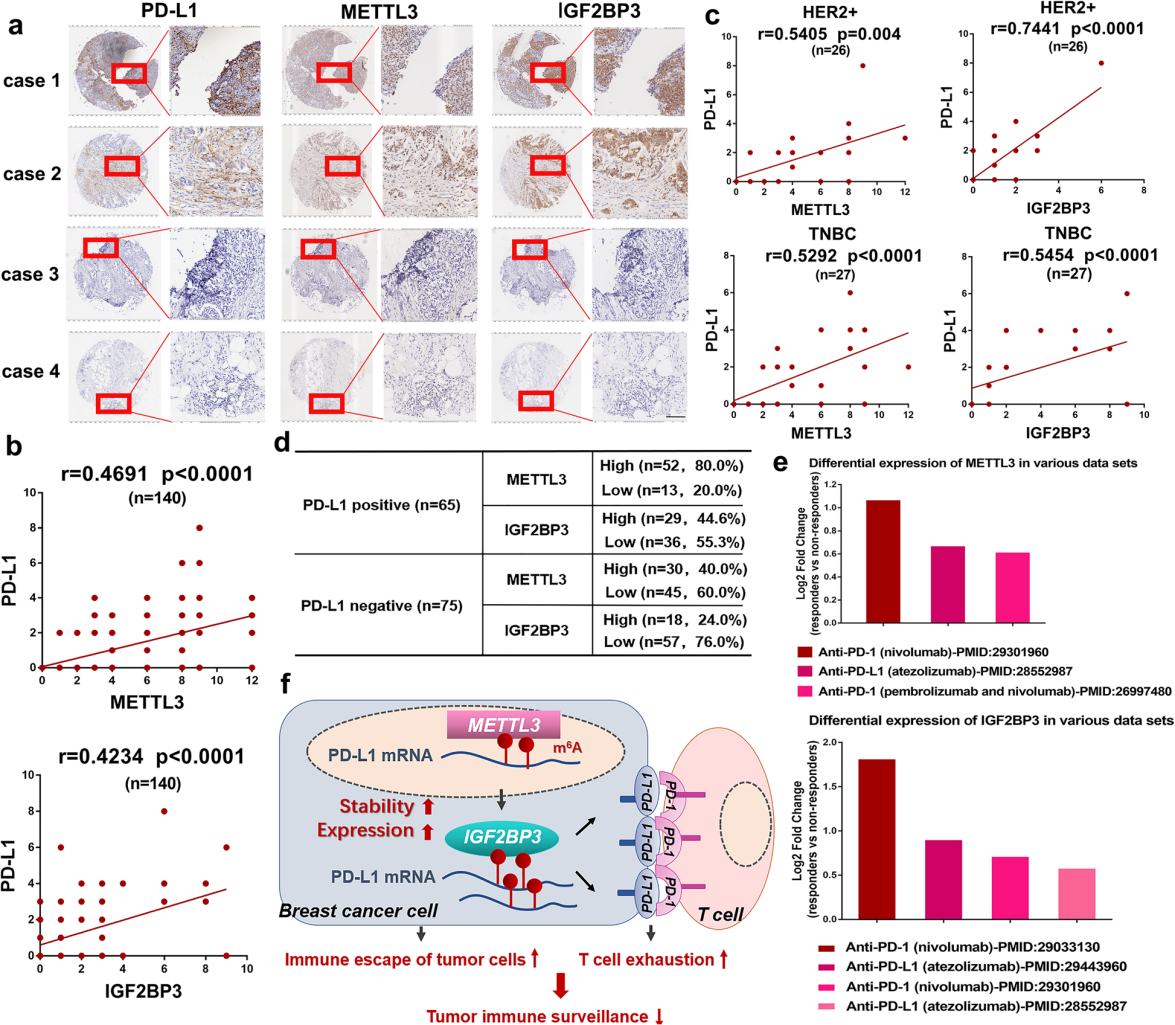
PD-L1 expression positively correlates with METTL3 and IGF2BP3 expression in breast cancer. **a** The expressions of PD-L1, METTL3 and IGF2BP3 were analyzed by IHC in a tissue microarray containing of 140 breast cancer tissues. Four Cases as representative IHC staining with positive- and negative-PD-L1 were shown. Scale bars, 100 μm. **b** The correlation of PD-L1 with METTL3 and IGF2BP3 in all breast cancer tissues (*n* = 140) were analyzed by IHC scores. Proportion scores were recorded as 0, 1, 2, 3, 4 corresponding to < 5%, 5–25%, 25–50%, 50–75%, and ≥ 75%. Intensity scores were recorded as 0, 1, 2, 3 corresponding to negative, weak, moderate, and strong staining. Finally, IHC scores was calculated as “proportion score × intensity score”. **c** The correlation between PD-L1 and METTL3 or IGF2BP3 were analyzed in HER2+ (*n* = 26) and TNBC (*n* = 27) subtypes. Spearman’s rank correlation test was used to analyze the *P* value. **d** Number of cases of METTL3 and IGF2BP3 were presented in two categories (PD-L1 positive and PD-L1 negative) in 140 tissues. **e** The differential expression of METTL3 or IGF2BP3 between responders and non-responders in cilnial data sets. The Y-axis represents the log2 Fold change values (responders vs. non-responders). **f** A schematic model illustrating the mechanism of METTL3/IGF2BP3-mediated N^6^-methyladenosine modification of PD-L1 mRNA in breast cancer

To determine the clinical correlation between PD-L1 and METTL3 or IGF2BP3, IHC staining was performed to verify the protein expression of METTL3, IGF2BP3, and PD-L1 using a tissue microarray consisting of 140 breast cancer tissues. We chose four patients as representatives including two cases of positive and two cases of negative PD-L1 expression (Fig. 6a). After evaluating the respective staining intensity scores, we found that, indeed, the PD-L1 expression in breast cancer was positively associated with the expression of METTL3 or IGF2BP3 (Fig. 6b). Next, we also analyzed the expression correlation in different subtypes of breast cancer. The results indicated that the correlation of METTL3 or IGF2BP3 with PD-L1 was higher in HER2+ (HER2 positive) and TNBC as compared to other subtypes (Fig. 6c, Additional file 6: Fig. S6a). Additionally, PD-L1-positive tissues expressed higher levels of METTL3 and PD-L1-negative tissues showed a concomitant decrease in IGF2BP3 expression, which suggested the existence of a METTL3-IGF2BP3-PD-L1 regulating axis in breast cancer (Fig. 6d). Next, we found that both METTL3 and IGF2BP3 expression levels were higher in TNBC and HER2+ subtypes which represented more aggressive phenotypes (Additional file 6: Fig. S6b). Additionally, due to the lack of access to validated data for breast cancer, we used TISIDB database (http://cis.hku.hk/TISIDB/index.php) [47] to evaluate whether METTL3 and IGF2BP3 showed differential expression in clinical data between responders and non-responders undergoing anti-PD-1/PD-L1 treatment. The results showed that tumors of the responders expressed higher levels of METTL3 (three data sets) and IGF2BP3 (four data sets) (Fig. 6e), which suggested that tumors with higher METTL3 or IGF2BP3 expression were likely to be sensitive towards anti-PD-1/PD-L1 immunotherapy. Collectively, these clinical sample data also verified that the expressions of METTL3 and IGF2BP3 were positively correlated with PD-L1 and indicated that PD-L1 could be a potential downstream target of the METTL3/IGF2BP3 axis in breast cancer.

## Discussion

PD-L1 is a major co-inhibitory immune-checkpoint protein and the PD1/ PD-L1 axis can inhibit the killing effect of cytotoxic T cells in the tumor microenvironment, further resulting in tumor immune escape [5, 6, 28]. Given the knowledge of these mechanisms, the microenvironment and immune-mediated factors in certain breast cancers have become significant for the development of treatment strategies [29]. Initially, breast cancer was not considered as an immunogenic tumor, however, recent studies show that aggressive triple-negative breast cancer, resistant to chemotherapy with poor prognosis, are immunogenic [29]; they are responsive to immunotherapy [30, 31]. Atezolizumab in combination with nab-paclitaxel is effective in unresectable, metastatic, or locally advanced TNBC, where the tumor is PD-L1-positive. These patients may thus benefit from immunotherapy [32]. However, objective responses to PD-L1 blockade therapy in breast cancer trials are not very encouraging [33]. Continual PD-L1 expression may affect its efficacy in clinical application. Therefore, further studies on the regulatory mechanisms of PD-L1 expression in breast cancer are needed. These could advance the current molecular understanding of immunoregulation and provide better immunotherapy strategies.

In this study, we found a critical role for m^6^A RNA modifications in the regulation of PD-L1 expression, stability, and T-cell-mediated killing in breast cancer. Thus, post-transcriptional modifications may be a promising therapeutic strategy for immunoregulation. N^6^-methyladenosine (m^6^A) modifications are prominent internal chemical modifications of RNA that are involved in multiple cellular activities, including RNA stability, protein translation, and molecular structure switching, which profoundly regulate several physiological processes and disease pathogenesis [18–21, 34–36]. Our results showed that m^6^A modification plays an important role in tumor immune evasion by upregulating PD-L1 expression and stability in breast cancer. First, using multidimensional sequencing technology, we identified PD-L1 as a potential direct downstream target of METTL3-mediated m^6^A alteration in breast cancer cells. The inhibition of METTL3 indeed led to PD-L1 downregulation, decreased binding and m^6^A levels, which was confirmed by qRT-PCR, MeRIP-qPCR, RIP-qPCR, and luciferase assays. We also evaluated the exact methylation level of each m^6^A site in the CDS sequence of PD-L1 mRNA at a single-nucleotide resolution using a new method for absolute quantification of m^6^A levels. Additionally, METTL3 knockdown could functionally improve T-cell killing and inhibit T-cell exhaustion to enhance immune evasion by downregulating PD-L1 expression. In addition to investigating the mechanism of PD-L1 m^6^A modification and in vitro cellular functions, we also analyzed the potential therapeutical effects of knocking down METTL3 in immunodeficient and immunocompetent mice models as compared to anti-PD-L1 treatment. The results suggested that silencing METTL3 eliminated the progression of xenograft tumors and increased the abundance of infiltrating immune cell types in the tumor microenvironment. Our results were similar to that of a previous study which shows that FTO depletion inhibits the expression of PD-L1 in colon cancer cells through m^6^A modification [43]. However, our study aimed at investigating the detailed specific molecular mechanisms underlying PD-L1 m^6^A modification and the impact of METTL3 on immunoregulation in breast cancer.

METTL3, through its methyltransferase activity, influences several biological processes and plays multiple roles in cancers. Previous studies suggest that diverse signals and pathways in cancers are regulated by METTL3, including cell proliferation, invasion, metastasis, and drug resistance [22–25, 37–42]. In breast cancer, METTL3 enhances the expression of HBXIP and induces positive feedback of HBXIP/let-7 g/METTL3/HBXIP signaling axis on cell proliferation [39]. In addition, METTL3 deficiency also influences macrophage reprogramming and enhances tumor progression in mouse models, thereby, eliminating the efficacy of PD-1 blockade treatment [26]; mRNA methylation mediated by METTL3/14 sensitizes pMMR-MSI-L colorectal cancer immunity to anti-PD-1 treatment by increasing STAT1 and IRF1 expression in an m^6^A-dependent manner [25]. Our study laid a strong case for a novel mechanism of METTL3 function as an immunomodulator in the tumor microenvironment of breast cancer, in addition to regulating tumor progression.

m^6^A reader proteins, such as YTHDF1/2/3, YTHDC1/2, and IGF2BP1/2/3, can bind to modified motifs of each target to exert diverse biological effects and influence the genetic information flow [44, 45]. The IGF2BP (IGF2BP1/2/3) family is especially pivotal for recognizing m^6^A modifications and regulates mRNA stabilization and translation. We identified that only IGF2BP3 could significantly decrease the expression and stability of PD-L1 in breast cancer cells. We investigated the role of IGF2BP3 as a reader of PD-L1 m^6^A methylation and through IGF2BP3-RIP analysis we found an enrichment of PD-L1 mRNA; this interaction was interrupted due to METTL3 deficiency. Furthermore, IGF2BP3 preferentially recognizes the m^6^A modification and influences METTL3-mediated regulation to prevent PD-L1 degradation. Correspondingly, we also found that IGF2BP3 knockdown was necessary to promote T cell-induced immune attack in breast cancer cells. Since the expression of IGF2BP3 could not be detected in the immunocompetent BALB/c mice, we used the TIMER [46] and TISIDB databases [47] for the analyses. The results indicated that IGF2BP3 expression was positively correlated with infiltration of CD8^+^, CD4^+^ T cells, and B cells in each of the breast cancer subtypes (Additional file 5: Fig. S5a, b), which was similar to the effect of PD-L1 (CD274). It has been reported that IGF2BP3, a well-known oncoprotein, is post-transcriptionally active and is involved in tumor growth, metastasis, survival, and chemo-resistance in the gastric, liver, and breast cancers and self-renewal and tumor initiation in cancer stem cells [48–50]. Our findings thus enriched the understanding of the function of m^6^A reader protein-mediated immunoregulation in breast cancer.

Additionally, we used the tissue microarray of breast cancer and found that METTL3, IGF2BP3, and PD-L1 were positively correlated by IHC staining. More importantly, the higher expression of METTL3 or IGF2BP3 was also found in patients who received PD-1/PD-L1 blockade therapy and seemed to show better responses. However, because of the limited clinical data, the efficacy of clinical trials in breast cancer could not be adequately captured in this study. Thus, further larger sample sizes are needed for clinical investigation and examination of in-detailed mechanisms underlying METTL3/IGF2BP3-induced antitumor immunity.

Taken together, the present study on METTL3/IGF2BP3-mediated N^6^-methyladenosine modification of PD-L1 mRNA and antitumor immunity provided a novel mechanism for m^6^A regulator-induced immunosuppression in breast cancer, which may have potential application as a novel therapeutic target.

## Conclusion

In conclusion, our study has illustrated the critical role of METTL3-mediated m^6^A modification in PD-L1 mRNA stabilization in an IGF2BP3-associated manner in breast cancer cells. Our findings broaden knowledge of the epi-transcriptional regulation mechanisms of PD-L1 expression and the functional value of m^6^A methyltransferase in tumor immune surveillance which may have important implications for new and efficient therapeutic strategies in the tumor immunotherapy.

## Supplementary Information

**Additional file 1: Fig. S1.** PD-L1 is a downstream target of METTL3. a RIP-seq demonstrated the METTL3 binding profile in PD-L1 gene. b PD-L1 mRNA expression was tested by qRT-PCR in several breast cancer cells.

**Additional file 2: Fig. S2.** METTL3 increases N^6^-methyladenosine modification and expression of PD-L1 mRNA. a Enrichment of METTL3 on PD-L1 mRNA was analyzed by RIP-qPCR in breast cancer cells compared to IgG. b-c The interaction between METTL3 and PD-L1 mRNA was analyzed by RIP-qPCR assay in MDA-MB-231 and HCC38 cells with overexpression or knockdown of METTL3. d-e The relative levels of m^6^A in PD-L1 were tested by MeRIP-qPCR in SK-BR-3 and HCC38 cells with knockdown or overexpression of METTL3. f The mRNA lifetime of PD-L1 transcripts in breast cancer cells with (shMETTL3) or without (sh-control) METTL3 silencing. g Schematic representation of experiment for absolute quantification of m^6^A modification. h-j The expression levels of PD-L1 were analyzed by qRT-PCR, western blot and flow cytometry in SK-BR-3 and HCC38 cells transfected with or wihthout sh-METTL3. *p < 0.05; **p < 0.01; ***p < 0.001.

**Additional file 3: Fig. S3.** IGF2BP3 mediates the mRNA expression of PD-L1 in m^6^A-dependent manner. a The mRNA level of PD-L1 was tested by qRT-PCR in MDA-MB-231 cell with knockdown of IGF2BP1 or IGF2BP2. b-d The expression levels of PD-L1 mRNA, protein and membrane were investigated by qRT-PCR, flow cytometry and western blot in SK-BR-3 cells. e Enrichment of IGF2BP3 on PD-L1 mRNA was analized by RIP-qPCR in breast cancer cells compared to IgG. f-g The interaction between IGF2BP3 and PD-L1 mRNA was analyzed by RIP-qPCR assay in SK-BR-3 cells. h The interaction between IGF2BP3 and PD-L1 mRNA with m^6^A mutation was detected by RIP-qPCR in breast cancer cells. i Enrichment of IGF2BP3 on PD-L1 mRNA was detected by RIP-qPCR assay in control and METTL3-knockdown cells. j PD-L1 mRNA levels were analyzed by qRT-PCR assay in SK-BR-3 cells after actinomycin D treatment. k The expression of PD-L1 protein was determined by western blot with transfection of indicated genes.*p < 0.05; **p < 0.01; ***p < 0.001; n.s., no significance.

**Additional file 4: Fig. S4.** METTL3/IGF2BP3-downregulated antitumor immunity in breast cancer cells. a The schematic illustrates the protocol for administration in B-NDG mice. b-c The volume of tumors were measured in the MDA-MB-231 cells-constructed xenograft models with indicated treatment. d The effect of METTL3 disruption on tumour growth was verified by luciferase activities assay in BALB/c peritonealtumor xenograft models. e The survival times were recorded and visualized using Kaplan-Meier survival curve. f CD3/ 4/ 8-positive cell number per high-power field (HPF) using immunohistochemistry (*n* = 3). g-h Volume and weight of tumors were determined in the subcutaneous-tumor with knockdown of METTL3. i CD3/ 4/ 8-positive cell number per high-power field (HPF) using immunohistochemistry (*n* = 3).

**Additional file 5: Fig. S5.** IGF2BP3 expression positively correlates with infiltration levels of immune cells in breast cancer. a Scatterplots of correlation between IGF2BP3 or PD-L1 expression and abundance of immune infiltration from TIMER database in breast cancer subtypes were shown. b IGF2BP3 expression had significant positive correlations with act-B Tcells/ CD8 T cells/ CD4 T cells, as with PD-L1.

**Additional file 6: Fig. S6.** The posotive correlation of PD-L1 with METTL3 and IGF2BP3. a The correlation between PD-L1 and METTL3 or IGF2BP3 in luminal A (*n* = 41) and luminal B (*n* = 38) subtypes were analyzed by Spearman’s rank correlation test. b The expression of METTL3 and IGF2BP3 in luminal A, luminal B, HER2+ and TNBC subtypes.

**Additional file 7: Table S1.** Sequences of primers and antibodies used in this study**. Table S2.** The list of down-modified genes in the intersection of epitranscriptomic microarray and MeRIP-seq.

## Data Availability

The datasets supporting the conclusions of this article are included within the article and its additional files.

## Abbreviations

m^6^A: N^6^-methyladenosine
METTL3: Methyltransferase-like 3
IGF2BP3: Insulin-like growth factor-2 mRNA-binding protein 3
MeRIP: Methylated RNA immunoprecipitation
RIP: RNA immunoprecipitation
DAA: 3-Deazaadenosine
IgG: Immunoglobulin G
CDS: Protein-coding transcripts region
ISO: Iso control antibody
TNBC: Triple-negative breast cancer
HER2 +: HER2-positive breast cancer
IHC: Immunohistochemistry
IL-2: Interleukin-2
PD-1: Programmed death receptor-1
PD-L1: Programmed death-ligand 1
IFN-γ: Interferonγ
TIM3: Hepatitis A virus cellular receptor 2
NR4A1: Nuclear receptor subfamily 4, group A, member 1
