# Supplementary figures and images for: METTL3/IGF2BP3 axis inhibits tumor immune surveillance by upregulating N6-methyladenosine modification of PD-L1 mRNA in breast cancer
Source: Mol Cancer. 2022 Feb 23;21:60. doi: 10.1186/s12943-021-01447-y (PMC8864846; doi:10.1186/s12943-021-01447-y)

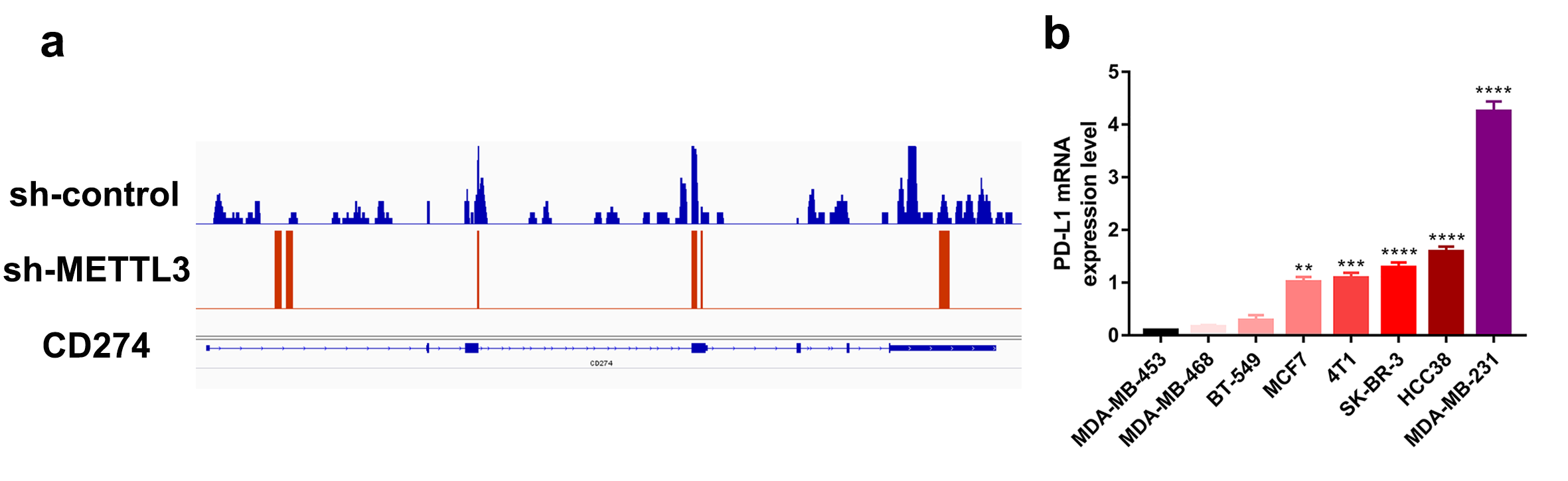

Supplement: Supplementary file 1 — Additional file 1: Fig. S1. PD-L1 is a downstream target of METTL3. a RIP-seq demonstrated the METTL3 binding profile in PD-L1 gene. b PD-L1 mRNA expression was tested by qRT-PCR in several breast cancer cells. [file 12943_2021_1447_MOESM1_ESM.tif]

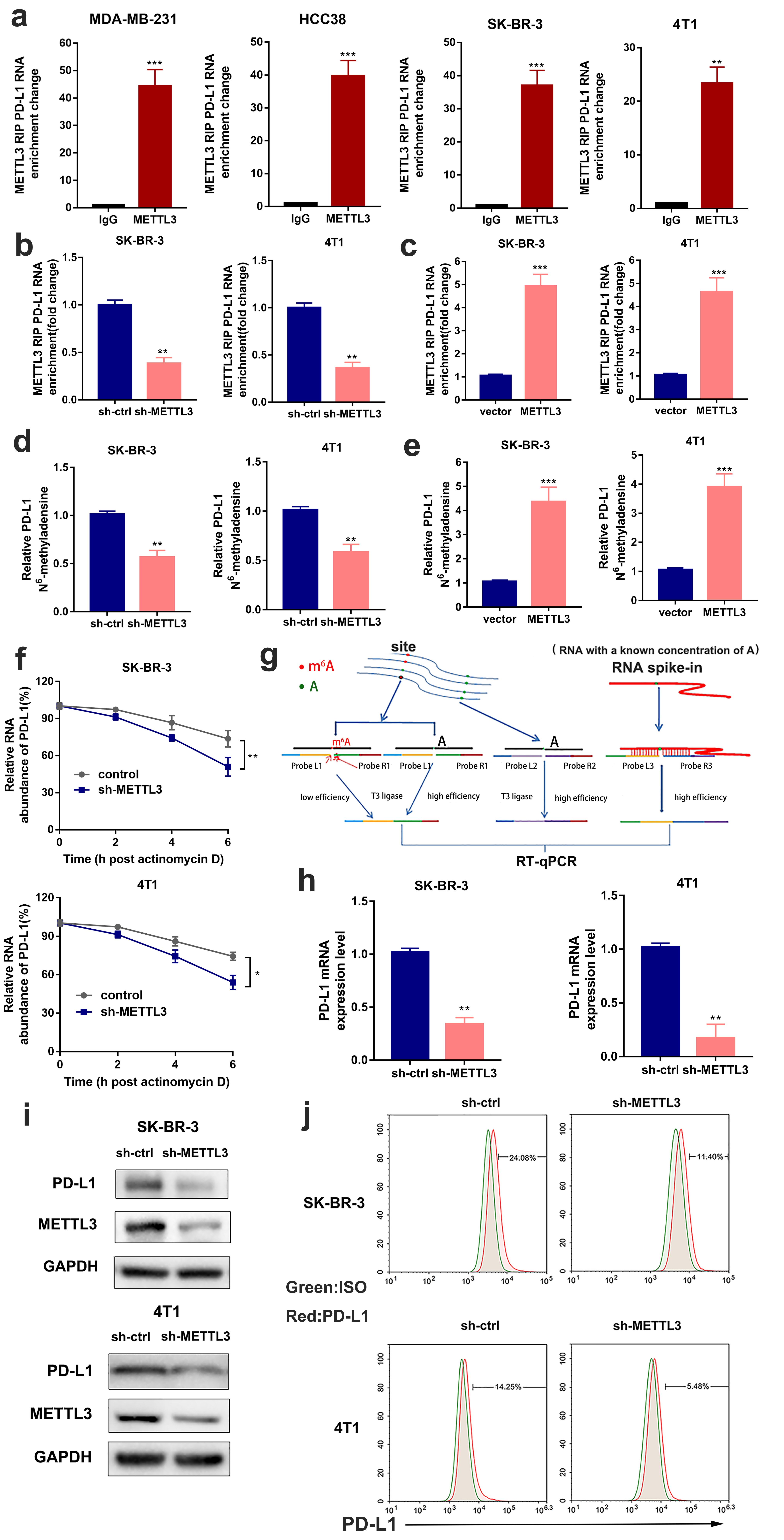

Supplement: Supplementary file 2 — Additional file 2: Fig. S2. METTL3 increases N6-methyladenosine modification and expression of PD-L1 mRNA. a Enrichment of METTL3 on PD-L1 mRNA was analyzed by RIP-qPCR in breast cancer cells compared to IgG. b-c The interaction between METTL3 and PD-L1 mRNA was analyzed by RIP-qPCR assay in MDA-MB-231 and HCC38 cells with overexpression or knockdown of METTL3. d-e The relative levels of m6A in PD-L1 were tested by MeRIP-qPCR in SK-BR-3 and HCC38 cells with knockdown or overexpression of METTL3. f The mRNA lifetime of PD-L1 transcripts in breast cancer cells with (shMETTL3) or without (sh-control) METTL3 silencing. g Schematic representation of experiment for absolute quantification of m6A modification. h-j The expression levels of PD-L1 were analyzed by qRT-PCR, western blot and flow cytometry in SK-BR-3 and HCC38 cells transfected with or wihthout sh-METTL3. *p < 0.05; **p < 0.01; ***p < 0.001. [file 12943_2021_1447_MOESM2_ESM.tif]

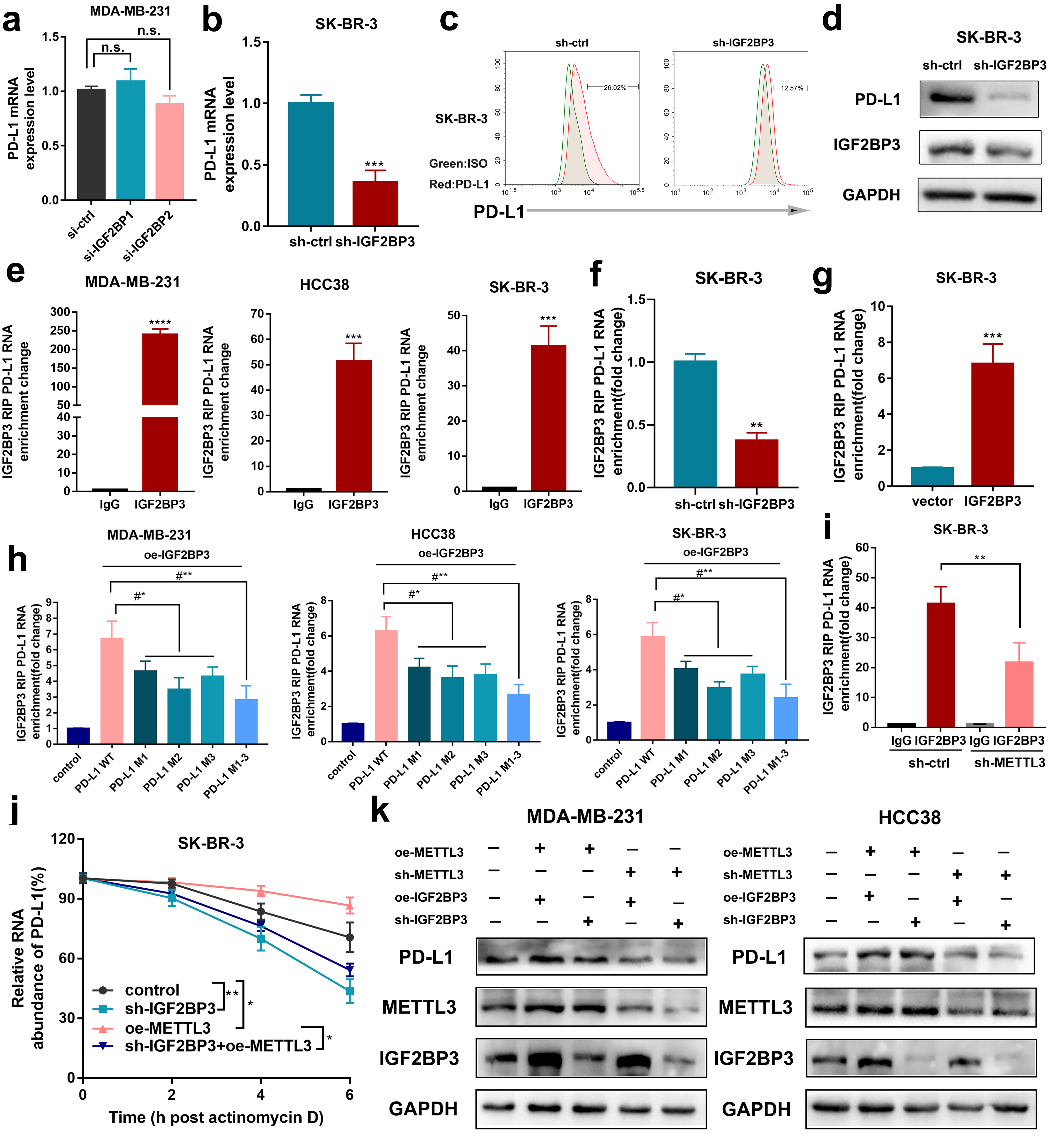

Supplement: Supplementary file 3 — Additional file 3: Fig. S3. IGF2BP3 mediates the mRNA expression of PD-L1 in m6A-dependent manner. a The mRNA level of PD-L1 was tested by qRT-PCR in MDA-MB-231 cell with knockdown of IGF2BP1 or IGF2BP2. b-d The expression levels of PD-L1 mRNA, protein and membrane were investigated by qRT-PCR, flow cytometry and western blot in SK-BR-3 cells. e Enrichment of IGF2BP3 on PD-L1 mRNA was analized by RIP-qPCR in breast cancer cells compared to IgG. f-g The interaction between IGF2BP3 and PD-L1 mRNA was analyzed by RIP-qPCR assay in SK-BR-3 cells. h The interaction between IGF2BP3 and PD-L1 mRNA with m6A mutation was detected by RIP-qPCR in breast cancer cells. i Enrichment of IGF2BP3 on PD-L1 mRNA was detected by RIP-qPCR assay in control and METTL3-knockdown cells. j PD-L1 mRNA levels were analyzed by qRT-PCR assay in SK-BR-3 cells after actinomycin D treatment. k The expression of PD-L1 protein was determined by western blot with transfection of indicated genes.*p < 0.05; **p < 0.01; ***p < 0.001; n.s., no significance. [file 12943_2021_1447_MOESM3_ESM.tif]

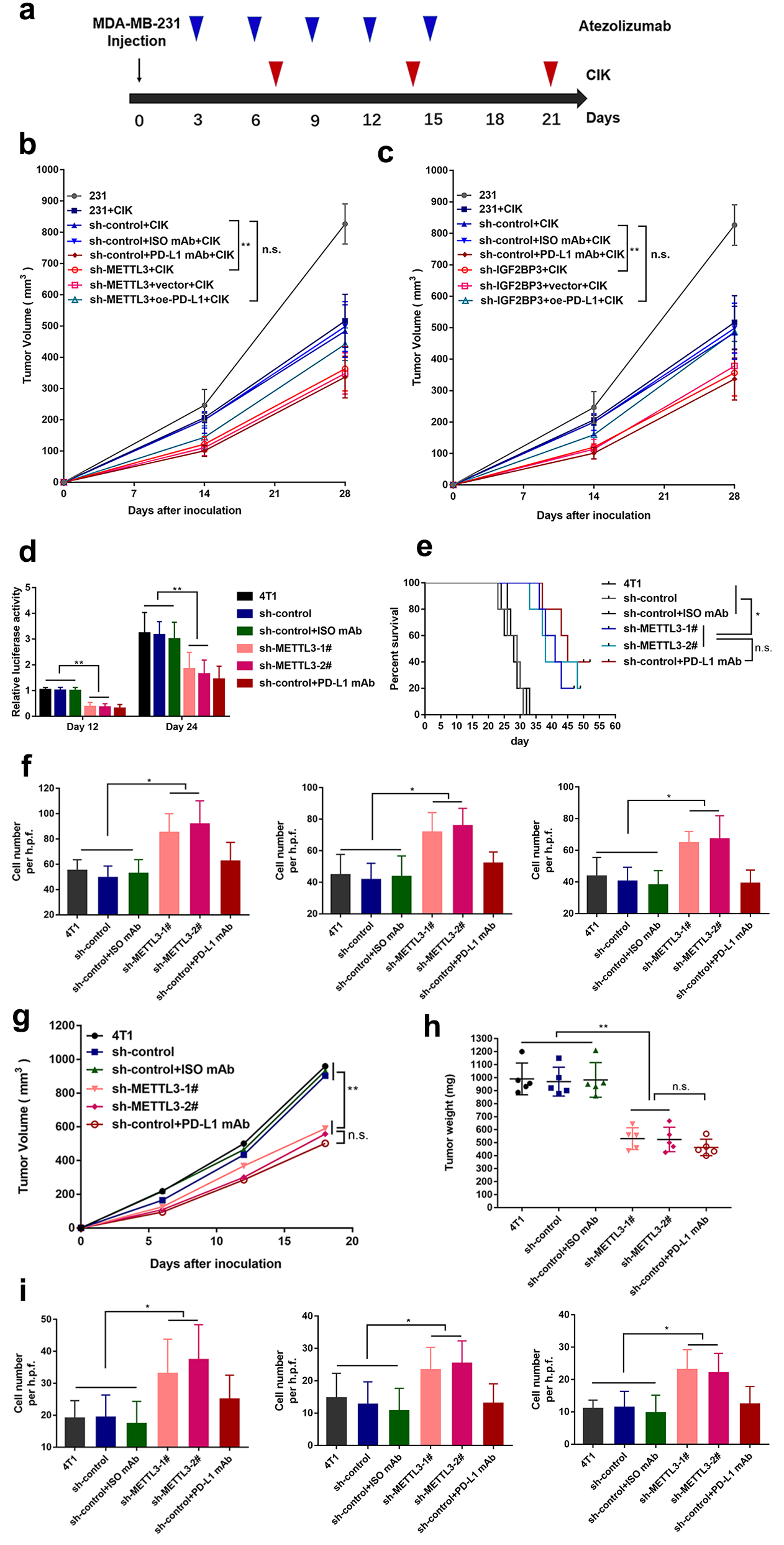

Supplement: Supplementary file 4 — Additional file 4: Fig. S4. METTL3/IGF2BP3-downregulated antitumor immunity in breast cancer cells. a The schematic illustrates the protocol for administration in B-NDG mice. b-c The volume of tumors were measured in the MDA-MB-231 cells-constructed xenograft models with indicated treatment. d The effect of METTL3 disruption on tumour growth was verified by luciferase activities assay in BALB/c peritonealtumor xenograft models. e The survival times were recorded and visualized using Kaplan-Meier survival curve. f CD3/ 4/ 8-positive cell number per high-power field (HPF) using immunohistochemistry (n = 3). g-h Volume and weight of tumors were determined in the subcutaneous-tumor with knockdown of METTL3. i CD3/ 4/ 8-positive cell number per high-power field (HPF) using immunohistochemistry (n = 3). [file 12943_2021_1447_MOESM4_ESM.tif]

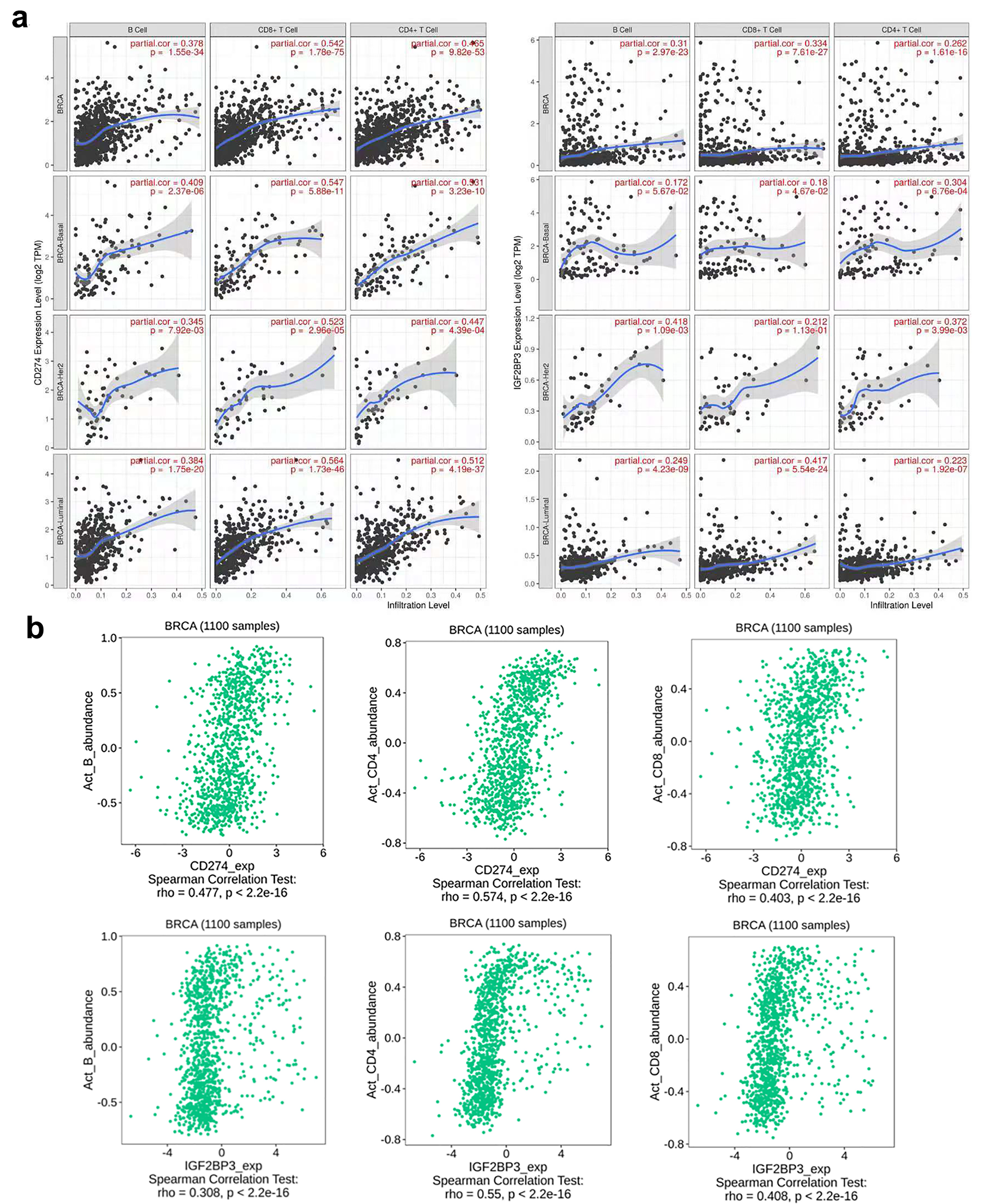

Supplement: Supplementary file 5 — Additional file 5: Fig. S5. IGF2BP3 expression positively correlates with infiltration levels of immune cells in breast cancer. a Scatterplots of correlation between IGF2BP3 or PD-L1 expression and abundance of immune infiltration from TIMER database in breast cancer subtypes were shown. b IGF2BP3 expression had significant positive correlations with act-B Tcells/ CD8 T cells/ CD4 T cells, as with PD-L1. [file 12943_2021_1447_MOESM5_ESM.tif]

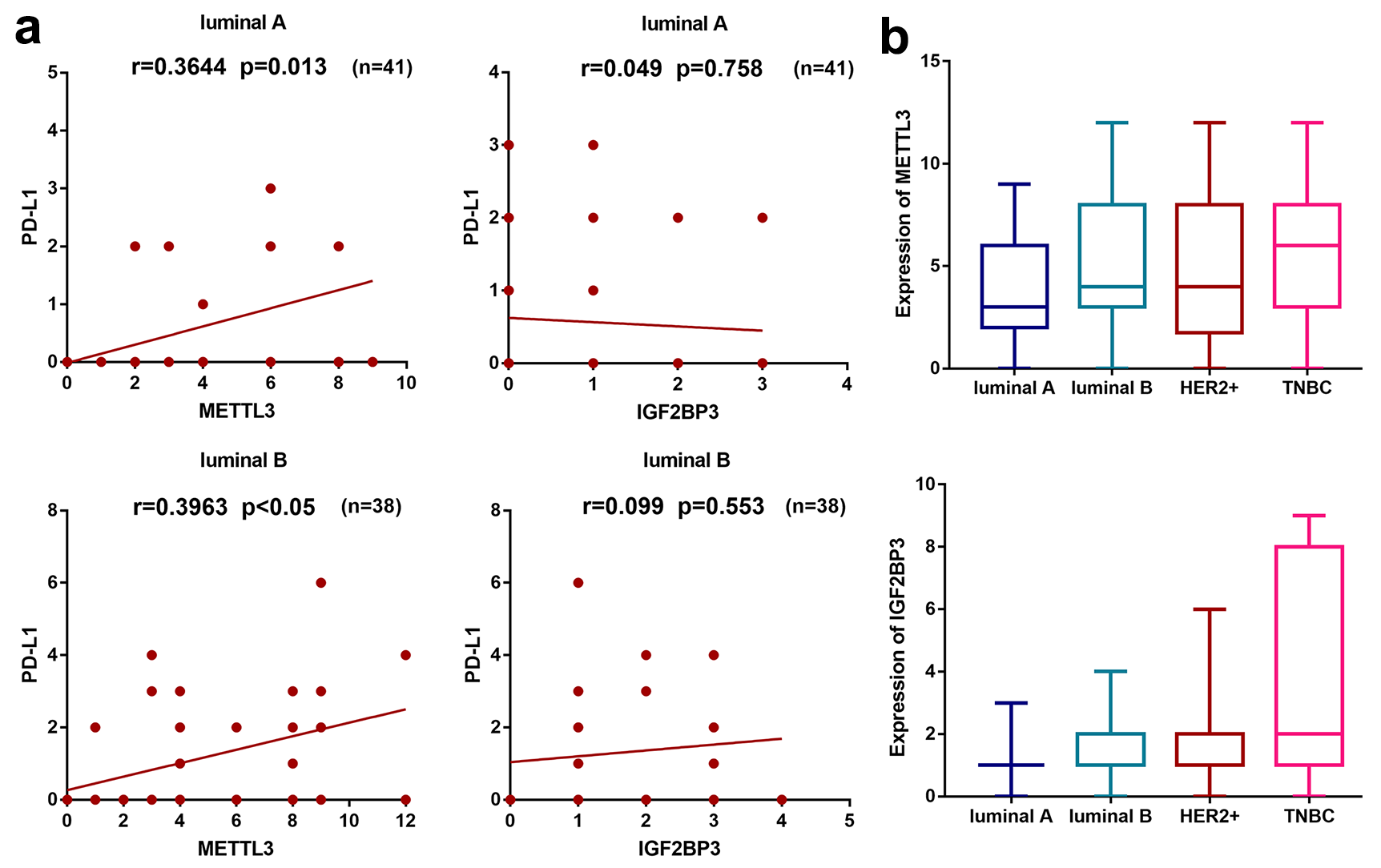

Supplement: Supplementary file 6 — Additional file 6: Fig. S6. The posotive correlation of PD-L1 with METTL3 and IGF2BP3. a The correlation between PD-L1 and METTL3 or IGF2BP3 in luminal A (n = 41) and luminal B (n = 38) subtypes were analyzed by Spearman’s rank correlation test. b The expression of METTL3 and IGF2BP3 in luminal A, luminal B, HER2+ and TNBC subtypes. [file 12943_2021_1447_MOESM6_ESM.tif]
