# Supplementary material for: METTL3/IGF2BP3 axis inhibits tumor immune surveillance by upregulating N6-methyladenosine modification of PD-L1 mRNA in breast cancer
Source: Mol Cancer. 2022 Feb 23;21:60. doi: 10.1186/s12943-021-01447-y (PMC8864846; doi:10.1186/s12943-021-01447-y)
Supplement: Supplementary file 7 — Additional file 7: Table S1. Sequences of primers and antibodies used in this study. Table S2. The list of down-modified genes in the intersection of epitranscriptomic microarray and MeRIP-seq. [file 12943_2021_1447_MOESM7_ESM.zip › Table S1.docx]

**Supplementary table 1. The primers used in this study.**

| Name | Sequence (5’-3’) |
| --- | --- |
| GAPDH-F | AAGGTGAAGGTCGGAGTCA |
| GAPDH-R | GGAAGATGGTGATGGGATTT |
| PD-L1-F | TGGCATTTGCTGAACGCATTT |
| PD-L1-R | TGCAGCCAGGTCTAATTGTTTT |
| METTL3-F | AGATGGGGTAGAAAGCCTCCT |
| METTL3-R | TGGTCAGCATAGGTTACAAGAGT |
| IGF2BP3-F | GCTCTATCAGTCGGTGCCATCATC |
| IGF2BP3-R | GCCTTGAACTGAGCCTCTGGTG |
| PD-L1-mouse-F | GGCCGAGGGTTATCCAGAAG |
| PD-L1-mouse-R | AAACATCATTCGCTGTGGCG |
| PD-L1-(peak1)-F^1^ | CTGTGAAAGTCAATGCCCCA |
| PD-L1-(peak1)-R^1^ | ACTTGATGGTCACTGCTTGTCC |
| PD-L1-(peak2)-F^1^ | CCTGAGGAAAACCATACAGC |
| PD-L1-(peak2)-R^1^ | GTGCTACACCAAGGCATAATAAG |
| RNA Spike in Probe^2^ | L5:po4 CCACTGACACGTAGAAAGTGGGCTGTGAAAGTAC  R5: TAGGACCAATATGCAACAACCCGACGTACCTrGrU |
| CD274-s1 Probe^2^ | L1:po4TCATGTTCAGAGGTGCAATACCTGGGAACATGGTAGT  R1: CCACCAATCCAGACAGAGTATTGCCTGACATGTCArGrU |
| CD274-s2 Probe^2^ | L2: CAGTTCATGTTCAGAACAACAACACCTTTAGCCCAAC  R2: TGTAGAGTCTGGATTTGCTGGATTCAGCCTGACATrGrU |
| CD274-s3 Probe^2^ | L3: CCTTTCATTTGGAGGACGTTTTCACCCATACCG  R3: CGAATGAACCATCGTGAGTGGAATTACCAAGTGArGrU |
| CD274-Control Probe^2^ | L4: TCACATCCATCATTCCGGAAATAGCAACCCATTGA  R4: GATGGCAAGAACTCTGTAACCCGATGCCACATTTrUrU |
| CD274-s1  PCR primer^2^ | F:ACTACCATGTTCCCAGGTATTG R:CCACCAATCCAGACAGAGTATT |
| CD274-s2  PCR primer^2^ | F:GTTGGGCTAAAGGTGTTGTTGT  R:TGTAGAGTCTGGATTTGCTGGAT |
| CD274-s3  PCR primer^2^ | F:CGGTATGGGTGAAAACGTC  R:CGAATGAACCATCGTGAGTG |
| CD274-Control  PCR primer^2^ | F:TCAATGGGTTGCTATTTCCG  R:GATGGCAAGAACTCTGTAACCC |
| RNA Spike in PCR primer^2^ | F:GTACTTTCACAGCCCACTTTCT  R:TAGGACCAATATGCAACAACC |
| Standard curve 1^2^ | \| F:TGTACGATTGGTTCAAAGGTCC \| \| --- \| \| R:CGCAAGATTTCAACTGGGTTC \| |
| Standard curve 2^2^ | \| F:GGTAAAGGTTCTTCGGCTGT \| \| --- \| \| R:TCTGGTGGCATCACTCATCT \| |
| Standard curve 3^2^ | \| F:ACTCCTGCTACCGTGTTGTG \| \| --- \| \| R:ACCTGGACCACTTGGAACG \| |
| Standard curve 4^2^ | \| F:GGTTGTCCTTGGGATTTTGT \| \| --- \| \| R:GGCATCTTTGTATGGGGTG \| |
| Standard curve 5^2^ | \| F:CATTTGGTGGTGGTAGACAG \| \| --- \| \| R:ATAATCAGGGTCAGGCACTT \| |
| Standard curve 6^2^ | \| F:GATTTGTCAAGCGTGAAAAAGA \| \| --- \| \| R:AGGTTGGGGAGTTGTAAGCA \| |
| PD-1-F | AAGGCGCAGATCAAAGAGAGCC |
| PD-1-R | CAACCACCAGGGTTTGGAACTG |
| TIM3-F | GACTCTAGCAGACAGTGGGATC |
| TIM3-R | GGTGGTAAGCATCCTTGGAAAGG |
| NR4A1-F | GGACAACGCTTCATGCCAGCAT |
| NR4A1-R | CCTTGTTAGCCAGGCAGATGTAC |

1: primers used in MeRIP-qpcr.

2: Primers used in Absolute quantification of m^6^A modification.

**Supplementary table 2. The core interference sequences of shRNAs.**

| Name | Sense |
| --- | --- |
| sh-control | TTCTCCGAACGTGTCACGT |
| sh-METTL3 | CCUGCAAGUAUGUUCACUA |
| sh-IGF2BP3 | GCTGAGAAGTCGATTACTA |
| sh-METTL3-1-mouse | CAAGGAAGAGTGCATGAAA |
| sh-METTL3-2-mouse | GAAAGGTCTTGGAGAGGTA |

**Supplementary table 3. Antibodies used in WB, IHC and IP.**

| Antibody (Item No.) | Specificity | Company |
| --- | --- | --- |
| GAPDH (D16H11) | WB (1:2000) | Cell Signaling Technology |
| METTL3 (E32FA) | WB (1:1000) | Cell Signaling Technology |
| IGF2BP3 (D6U2N) | WB (1:1000) | Cell Signaling Technology |
| PD-L1-human (E1L3N) | WB (1:1000) | Cell Signaling Technology |
| PD-L1-mouse (D4H1Z) | WB (1:1000) | Cell Signaling Technology |
| CD3 (ab16669) | IHC (1:150) | Abcam |
| CD4 (ab133616) | IHC (1:200) | Abcam |
| CD8 (ab209775) | IHC (1:200) | Abcam |
| METTL3 (ab195352) | IP (5 ug/per test) | Abcam |
| IGF2BP3 (03-198) | IP (5 ug/per test) | Millipore |
